# Three-dimensional periodic supramolecular organic framework ion sponge in water and microcrystals

**DOI:** 10.1038/ncomms6574

**Published:** 2014-12-03

**Authors:** Jia Tian, Tian-You Zhou, Shao-Chen Zhang, Shaul Aloni, Maria Virginia Altoe, Song-Hai Xie, Hui Wang, Dan-Wei Zhang, Xin Zhao, Yi Liu, Zhan-Ting Li

**Affiliations:** 1 Collaborative Innovation Center of Chemistry for Energy Materials (iChEM), Department of Chemistry, Fudan University, 220 Handan Road, Shanghai 200433, China; 2 Laboratory of Synthetic and Self-Assembly Chemistry for Organic Functional Molecules, Shanghai Institute of Organic Chemistry, Chinese Academy of Sciences, 345 Lingling Lu, Shanghai 200032, China; 3 The Molecular Foundry, Lawrence Berkeley National Laboratory, One Cyclotron Road, Berkeley, California 94720, USA

## Abstract

Self-assembly has emerged as a powerful approach to generating complex supramolecular architectures. Despite there being many crystalline frameworks reported in the solid state, the construction of highly soluble periodic supramolecular networks in a three-dimensional space is still a challenge. Here we demonstrate that the encapsulation motif, which involves the dimerization of two aromatic units within cucurbit[8]uril, can be used to direct the co-assembly of a tetratopic molecular block and cucurbit[8]uril into a periodic three-dimensional supramolecular organic framework in water. The periodicity of the supramolecular organic framework is supported by solution-phase small-angle X-ray-scattering and diffraction experiments. Upon evaporating the solvent, the periodicity of the framework is maintained in porous microcrystals. As a supramolecular ‘ion sponge’, the framework can absorb different kinds of anionic guests, including drugs, in both water and microcrystals, and drugs absorbed in microcrystals can be released to water with selectivity.

## Abstract (web summary)

The construction of soluble periodic supramolecular three-dimensional networks is challenging. Here, the authors use an encapsulated dimerization strategy to direct the assembly of a periodic three-dimensional supramolecular organic framework and evaluate its absorption properties.

## Graphical abstract

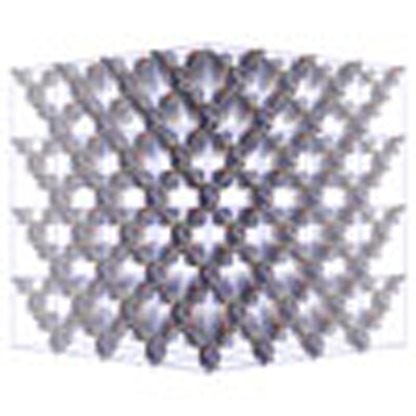

In the last four decades, an enormous body of research has been amassed on the topics of supramolecular chemistry and self-assembly^1^,^2^. Recently, considerable effort has been devoted to crystal engineering^3^ and supramolecular polymers^4^,^5^ for better control of bonding and ordering. By utilizing coordination or hydrogen bonding as driving forces, a large number of supramolecular crystalline frameworks^6^, including a hydrogen bonding-stabilized supramolecular organic framework (SOF)^7^, have been constructed, many of which generate channels that allow for gas, solvent or guest absorption^7^,^8^,^9^. However, none of these crystalline frameworks can maintain their periodicity in solution. Supramolecular polymers^4^,^5^,^10^,^11^,^12^,^13^,^14^,^15^,^16^,^17^,^18^, particularly these based on tetrahedral building blocks^19^,^20^,^21^,^22^, can produce three-dimensional (3D) cross-linked networks in solution. However, these networks generally lack periodicity in a 3D space. To the best of our knowledge, there are no prior examples of solution-phase 3D periodic supramolecular networks, although a solution-phase two-dimensional (2D) periodic supramolecular framework had been created by us^23^. The past two decades have also witnessed great advance in the design of 3D metal–organic frameworks^24^,^25^ and covalent organic frameworks based on strong coordination or covalent bonds^26^,^27^. Currently, such hard porous solid-state architectures have been vigorously studied for various functions or applications^28^,^29^, in particular, in gas storage^30^,^31^, absorption and separation^32^,^33^, recognition and sensing^34^,^35^, biomedical science^36^,^37^,^38^ and catalysis^39^,^40^,^41^. If self-assembly can be shown to drive the formation of periodic supramolecular networks in a 3D space in solution, rational design of molecular building blocks should lead to the construction of a variety of soluble ordered porous frameworks and thus offer new opportunities for the generation of advanced soft materials.

Herein, we report the first example of solution-phase self-assembly of a 3D periodic porous SOF in water, utilizing the encapsulation of cucurbit[8]uril (CB[8]) for two 4-(4-methoxyphenyl)pyridin-1-ium (PP) units. The periodic SOF not only exists in solution, but also can be maintained in the solid state. We further demonstrate that the porous framework, as a family of 3D-ordered cationic supramolecular polyelectrolyte, can attract a variety of anionic organic molecules, including dyes, drugs, peptides, nucleic acids and poly(amidoamine) (PAMAM) dendrimers, in both solution and the solid state, and the attracted drugs can be released to water selectively in acidic medium.

## Results

### The design of target and control molecules

**Figure 1:**
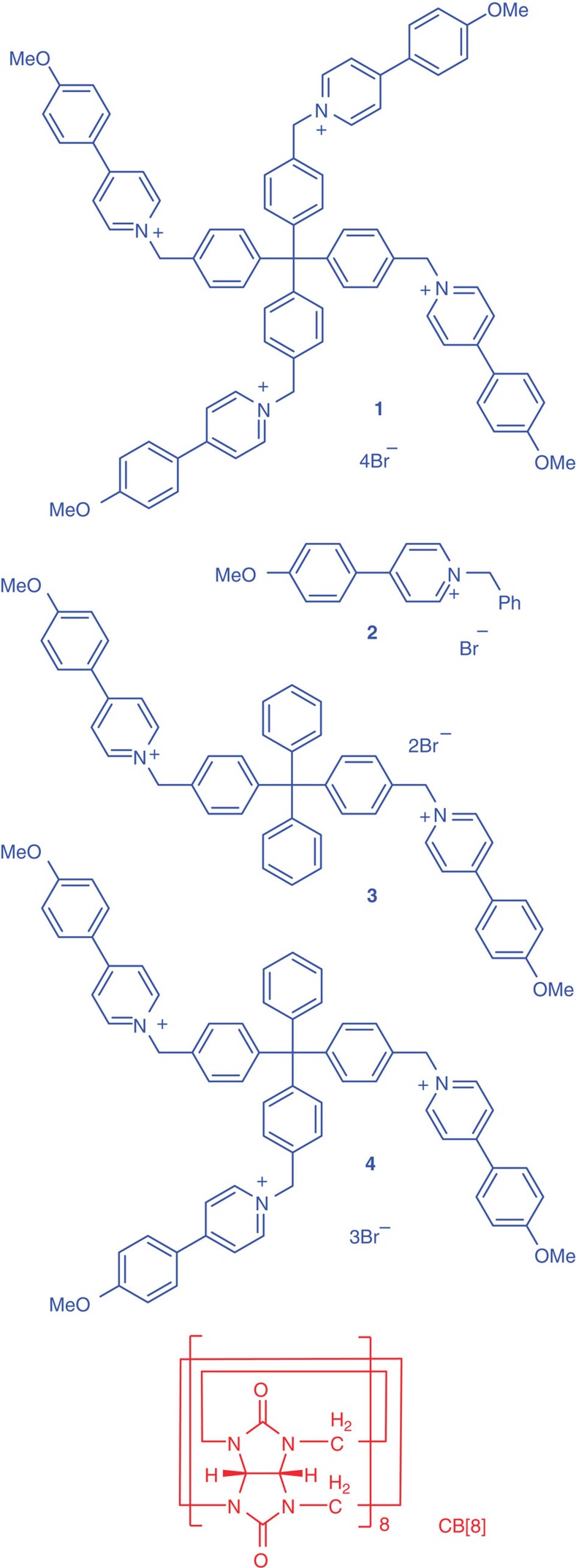
Compounds used in this study. The structures of compounds **1**–**4** and CB[8].

Pioneering work by Kim *et al.*^42^ established that CB[8] stabilizes the charge-transfer interaction between two different aromatic units in aqueous media. Recently, Zhao reported that CB[8] also enhances the homodimerization of 4-arylpyridin-1-ium units through hydrophobicity^43^. We thus designed and prepared compound **1** for assembling 3D SOF by utilizing the 1:2 binding motif between CB[8] and 4-(4-methoxyphenyl)pyridin-1-ium (PP) unit (Fig. 1). Three PP-containing compounds **2**–**4** were prepared as controls (Fig. 1).

### Spectroscopic and and mass spectrometric studies

**Figure 2:**
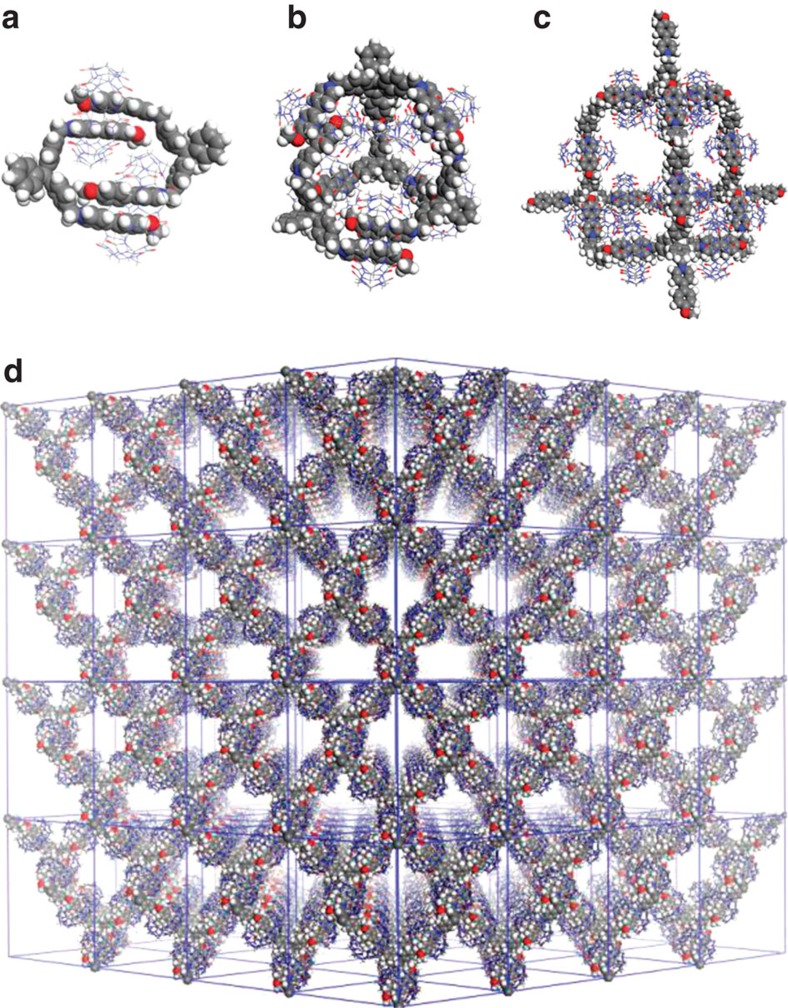
Self-assembled patterns. (**a**) Model of the 2+2 macrocycle **3**_2_·CB_2_ (**Com-D**). (**b**) Model of the 4+6 capsule **4**_4_·CB_6_ (**Com-Tri**). (**c**,**d**) Models of one adamantane-shaped unit and the 3D supramolecular organic framework (**1**_*n*_·CB_2*n*_, **Com-Tetra**).

The binding of the PP units of **1**–**4** by CB[8] in D_2_O was first studied by ^1^H nuclear magnetic resonance (NMR) spectroscopy (Supplementary Figs 1–4). For the 2:1 solution of **2** (8.0 mM) and CB[8], at 25 °C, the signals of the β-protons of the pyridinium unit and the protons of the connected benzene unit disappeared, while other signals became broader compared with those of **2** in the absence of CB[8]. Reducing the temperature to 5 °C led to the splitting of several aromatic proton signals, although the signals were all broad. These observations suggest that two complexation patterns, that is, the head-to-tail and head-to-head stacking of **2** within the cavity of CB[8], existed, although the crystal structures of the 2:1 complexes of two model molecules with CB[8] exhibited the head-to-tail stacking pattern^43^. The ^1^H NMR spectrum of the 1:1 solution of **3** (4.0 mM) and CB[8] indicated the formation of one major species and a minor one, which were assigned to the two isomers of a 2+2 macrocyclic complex **3**_2_·CB_2_ (**Com-D**, Fig. 2a) that coalesced at high temperature (Supplementary Fig. 3). Dynamic light-scattering (DLS) experiments for the 1:1 solution of **3** and CB[8] revealed that the hydrodynamic diameter (*D*_H_) of the complex formed by them did not change at elevated temperature (Supplementary Fig. 21e–g). This observation is consistent with the above ^1^H NMR experiments at high temperature, because the proposed isomers of **COM-D** were expected to possess comparable size. The formation of such 2+2 complex was further confirmed by the ionic peak of [**3**_2_·CB_2_-3Br]^3+^ at *m*/*z*=1,389.4648 (calculated value: 1,389.4608) from the electrospray ionization mass spectrometry (Supplementary Fig. 5). The ^1^H NMR spectrum of the 2:3 solution of **4** (2.7 mM) and CB[8] gave one set of signals associated with encapsulated **4**, and the addition of more CB[8] did not cause the signals to shift or the appearance of new signals. These signals were tentatively assigned to **4** in a stable and symmetric 4+6 capsular complex **4**_4_·CB_6_ (**Com-Tri**, Fig. 2b), because the modelled size of this complex matched the *D*_H_ of the species measured by DLS (vide infra). In contrast, the ^1^H NMR spectrum of the 1:2 solution of **1** (2.0 mM) and CB[8] displayed very broad signals, which remained unchanged at higher temperature or with more equivalent of CB[8]. These observations implied the formation of a more complicated supramolecular structure **1**_n_·CB[8]_2n_ (**Com-Tetra**, Fig. 2c). The 2:1 binding stoichiometry of the PP units of **1**–**4** and CB[8] was further confirmed by Job’s plots using fluorescence spectroscopy (Supplementary Figs 6–9). Using the competition ^1^H NMR method reported by Isaacs^44^, we determined the apparent association constant (*K*_a_) of the 2:1 complex between the PP unit of **2**, **3**, **4** and **1** and CB[8] to be 1.1 × 10^11^, 9.4 × 10^11^, 5.4 × 10^12^ and 1.3 × 10^14^ M^−2^, respectively, in 50 mM CD_3_CO_2_Na-buffered D_2_O (pD=4.74). The fact that the values increased substantially in the order of **2**<**3**<**4**<**1** excluded the formation of disordered supramolecular polymers by **3**, **4** and **1** because the encapsulation in their systems exhibited considerable positive cooperativity. For **4** and **1**, the results may also be rationalized by considering the formation of an ordered 2D or 3D supramolecular network^23^, which is expected to enhance the stability of the 2:1 complex. However, DLS experiments, which gave a small *D*_H_ of 4.0 nm for the mixture of **4** (vide infra), excluded the possibility of forming a 2D network by **4**.

2D ^1^H NMR diffusion-ordered spectroscopic (DOSY) experiments were performed for the solution of the four mixtures ([PP]/[CB[8]]=2, [PP]=4.0 mM) in D_2_O (ref. 45). In all four spectra, the signals of the complexes gave rise to the nearly identical diffusion coefficient (*D*), which was 2.5 × 10^−10^, 1.7 × 10^−10^, 1.4 × 10^−10^ and 4.8 × 10^−11^ m^2^ s^−1^ (Supplementary Figs 10–14), respectively. The notably lower *D* values of complexes **Com-D** and **Com-Tri**, compared with that of **Com-M**, indicated the formation of larger aggregates. The value of **Com-Tetra** was considerably lower than that of **Com-Tri**, suggesting that it produced even larger supramolecular aggregates. From DLS experiments in water conducted for the four mixtures at [PP]=6.0 mM, we determined the *D*_H_ values of the four mixtures to be 1.4, 2.9, 4.0 and 91.3 nm, respectively (Supplementary Figs 21a–d), the order of which was in agreement with the above DOSY results. The *D*_H_ values of **Com-D** and **Com-Tri** were pronouncedly larger than that of **Com-M** and reasonably matched with the calculated sizes of their respective 2+2 macrocycle (ca. 3.0 nm) and 4+6 capsule (ca. 4.1 nm). The result also excluded the formation of large extended networks by **Com-Tri**^23^. Remarkably, the *D*_H_ of **Com-Tetra** was >20 times larger than that of **Com-Tri**, which clearly indicated the formation of a large complex in the mixture of **1** and CB[8]. The *D*_H_ value for the solution of **Com-Tetra** also increased with the concentration and reached 100.5 nm at [PP]=8.0 mM (Supplementary Fig. 22). Further increase of the concentration resulted in the formation of a hydrogel (Supplementary Fig. 38). Similar concentration dependence was not observed for **Com-M**, **Com-D** and **Com-Tri**, which was consistent with that they just produced small aggregates.

### The characterization of 3D supramolecular structures

An ideal 3D supramolecular network formed by **Com-Tetra** would resemble the 3D net of diamond. The central carbon atom of **1** is the vertex of the net, and CB[8] locks two PP units of neighbouring molecules by holding them in its cavity. We then simulated the crystal structure of the 3D supramolecular network from the diamond topology using Materials Studio 5.0 (ref. 46), on the basis of the crystal structure parameters of the 2:1 complex of 4-(4-methoxyphenyl)pyridin-1-ium and CB[8]^43^,^47^. The obtained structure is shown in Fig. 2d. Considering the tetrahedral feature of compound **1** and the growth of the framework occurred in the 3D space, we conjecture that the aggregates formed in solution should be roughly spherical.

**Figure 3:**
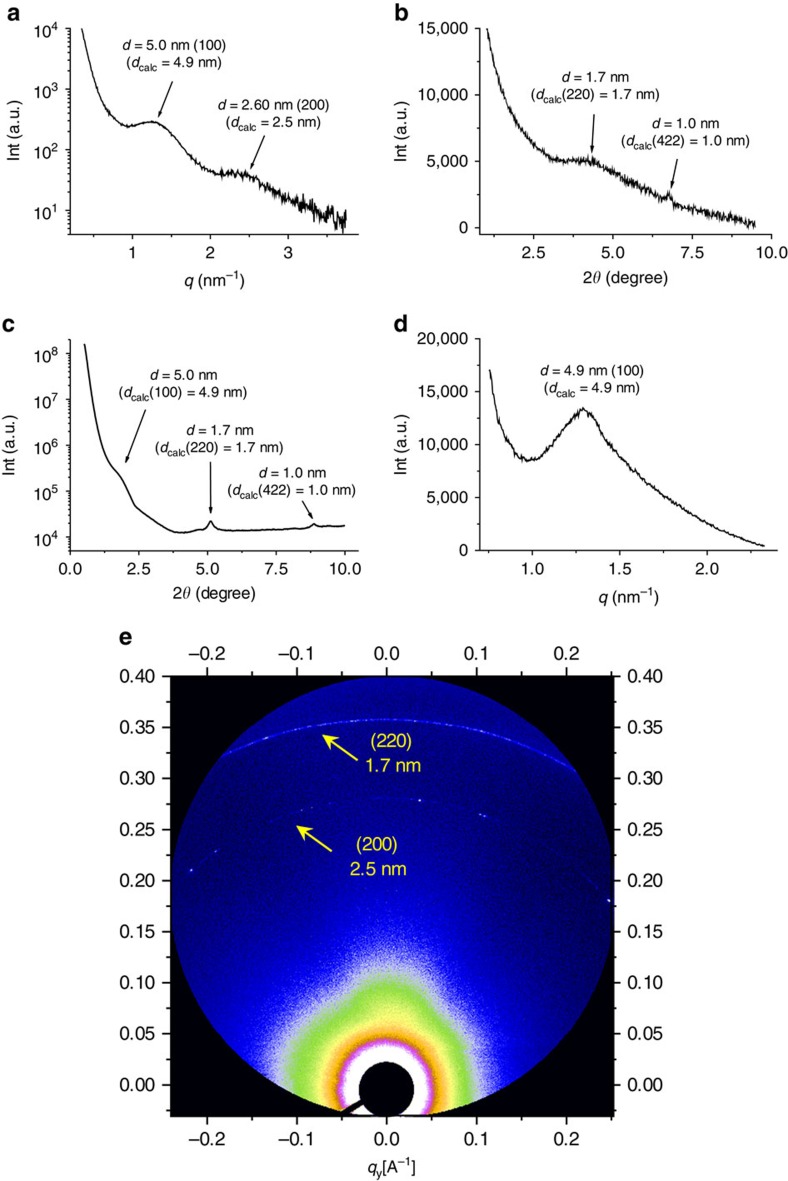
Solution- and solid-phase SAXS and X-ray diffraction profiles. (**a**) Solution-phase synchrotron small-angle X-ray-scattering profile of the solution of **Com-Tetra** ([PP]=8 mM) in water. (**b**) Solution-phase synchrotron X-ray diffraction (XRD) profile in water. (**c**) XRD profile of the solid sample obtained by evaporating the solution of **Com-Tetra** in water. The peak value was attributed by choosing the position that is highest above the straight line defined by the two saddle points of the broad peak. (**d**) Small-angle synchrotron X-ray-scattering profile of **Com-Tetra** of the solid sample. (**e**) Two-dimensional synchrotron X-ray-scattering profile of **Com-Tetra** of the solid sample.

To investigate the solution-phase periodicity of the 3D supramolecular network formed by **Com-Tetra**, we first carried out a small-angle X-ray-scattering (SAXS) experiment for the solution in water, which revealed a broad but clear peak corresponding to the d-spacing around 5.1 nm (Supplementary Fig. 23). This spacing matched well with the calculated {100} spacing (4.9 nm) of the modelled network, providing the first evidence for the periodicity of the 3D SOF in solution. When the solution was subjected to synchrotron radiation X-ray scattering, a much stronger peak corresponding to the d-spacing of 5.0 nm was observed, together with another broad peak with d-spacing of ca. 2.6 nm (Fig. 3a). This peak could be assigned to the {200} spacing, which has a calculated value of 2.5 nm. These results further confirmed the periodicity of the 3D SOF in solution. Synchrotron X-ray diffraction studies of the same solution also revealed a broad peak around 1.7 nm and a relatively sharp peak around 1.0 nm (Fig. 3b), which are again in agreement with the expected spacing (1.7 nm) of the {220} face and the spacing (1.0 nm) of the {422} face of the 3D SOF. The differences between the calculated and measured d-spacing may indicate solvent-induced expansion, and reflect the dynamic feature of the supramolecular frameworks in solution. The broad peaks in Fig. 3a,b may also reflect this dynamic feature.

**Figure 4:**
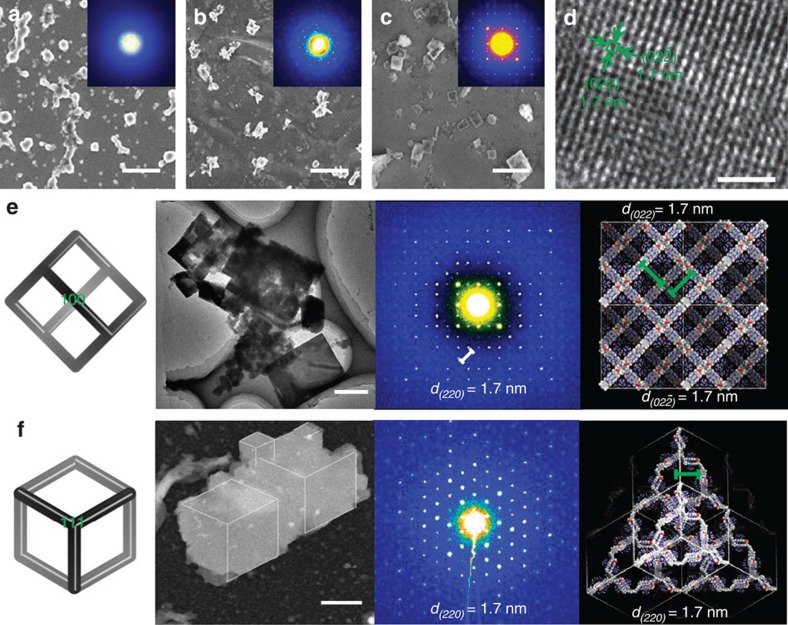
Electron microscopy and SAED. Scanning electron microscope (SEM) images, high-resolution TEM image and SAED pattern with model illustration. (**a**–**c**) SEM images of **Com-Tetra** recorded right after evaporation of the solvent, and after standing for 3 and 14 days (bar width=1 μm). The images highlighted microcrystallization of the sample. Inset: SAED patterns showing the different ordering at different time. (**d**) High-resolution TEM image of the periodic porous pattern of the microcrystal (bar width=10 nm). (**e**) Schematic illustration and Cryo-TEM (left two images of panel, bar width=0.5 μm), and SAED pattern and its model illustration (right two images of panel) of the microcrystals with the reciprocal lattice observed for the {100} facet, which showed foursquare order. (**f**) Schematic illustration and Cryo-TEM (left two images of panel, the white lines in the TEM image are eyeguides (bar width=0.3 μm, Supplementary Fig. 39), and SAED pattern and its model illustration (right two images of panel) of the microcrystal with the reciprocal lattice for the {111} facet, which showed hexagonal order.

During slow evaporation under ambient temperature, the above solution first turned into a hydrogel, and then slowly solidified due to further loss of the solvent. Scanning electron microscope images revealed that the solidification was accompanied with microcrystallization of the sample, which was evidenced by selected area electron diffraction (SAED) experiments (Fig. 4a–c, inset). The X-ray diffraction profile of the microcrystals exhibited three peaks centred at 5.0, 1.7 and 1.0 nm (Fig. 3c), respectively, which matched well with the {100}, {220} and {422} spacings of the modelled 3D framework (Fig. 2d). This result supported that the periodicity of the 3D SOF not only existed in solution, but also could be maintained in the solid state. Since the 100 peak in Fig. 3c was very weak, we further performed small-angle synchrotron X-ray scattering of **Com-Tetra** of the solid sample (Fig. 3d). A much stronger peak centred at 4.9 nm, corresponding to the {100} spacing, was exhibited. Two-dimensional synchrotron X-ray scattering was also carried out for the solid sample, which revealed two scattering peaks with d-spacings of 2.5 and 1.7 nm (Fig. 3e), which again matched well with the calculated values of the {200} and {220} spacings. High-resolution transmission electron microscopy (TEM) of the microcrystal revealed periodic porosity with 1.7-nm spacing (Fig. 4d), which also matched well with the simulated value (1.7 nm) for the {220} face. Thermogravimetric analysis showed that the supramolecular microcrystals were stable at a high temperature of 300 °C (Supplementary Fig. 40).

On the basis of the X-ray diffraction and TEM results and the parameters of the single crystal of the 1:2 complex of CB[8] and 1-methyl-4-(4-methoxyphenyl)pyridin-1-ium, we could determine the cubic unit-cell metrics (*a*=*b*=*c*=49.2 Å, *α*=*β*=*γ*=90°). SAED patterns of the microcrystals with the reciprocal lattice observed for the {100} and {111} facets showed high foursquare and hexagonal order, respectively (Fig. 4e,f), which further confirmed the cubic unit cell of the microcrystals. The SAED pattern, that is, 1.7 nm for {220} lattice spacing, matched well with the simulated datum (1.7 nm). Elemental mapping analysis for the microcrystals also confirmed the compositions of the C, N, O and Br elements (Supplementary Fig. 24).

### Absorption studies

**Figure 5:**
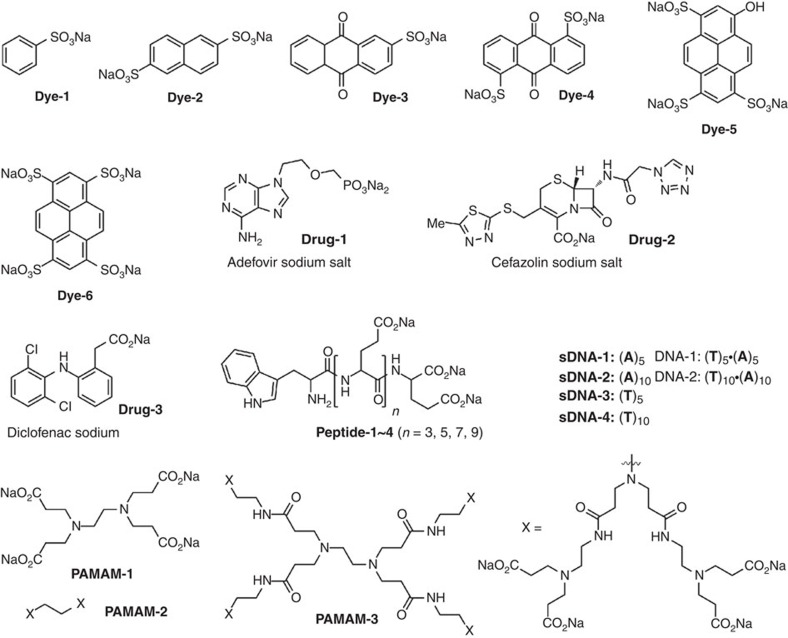
Guest molecules. Structures of guests **Dye-1~6**, **Drug-1~3**, **Peptide-1~4**, **sDNA-1~4**, **DNA-1~2** and dendrimers **PAMAM-1~3**.

The calculated void volume of the 3D SOF based on the above model was about 77%, which suggested high porosity. The pore aperture, defined by the six CB[8] units in one self-assembled macrocycle adopting a cyclohexane-like chair conformation, was calculated to be about 2.1 nm, while the size defined by the adamantane-like self-assembled cage was ever larger. Given the polycationic feature of this 3D framework, we envisioned that it should be able to attract organic anionic guests in solution by electrostatic interactions. We thus evaluated its inclusion property towards 20 different anionic guests of varying shape and size (Fig. 5), including dyes, drugs, peptides, sDNA and DNA and dendrimers. A systematic investigation of guest-induced fluorescent quenching of the PP unit was then performed, which allowed us to evaluate the inclusion capacity of 3D SOF against the other three discrete PP/CB[8] supramolecular complexes (Supplementary Figs 27–29). For comparison, all the experiments were carried out for the four mixtures by keeping the 2:1 molar ratio of the PP unit (0.02 mM) and CB[8] (0.01 mM).

**Figure 6:**
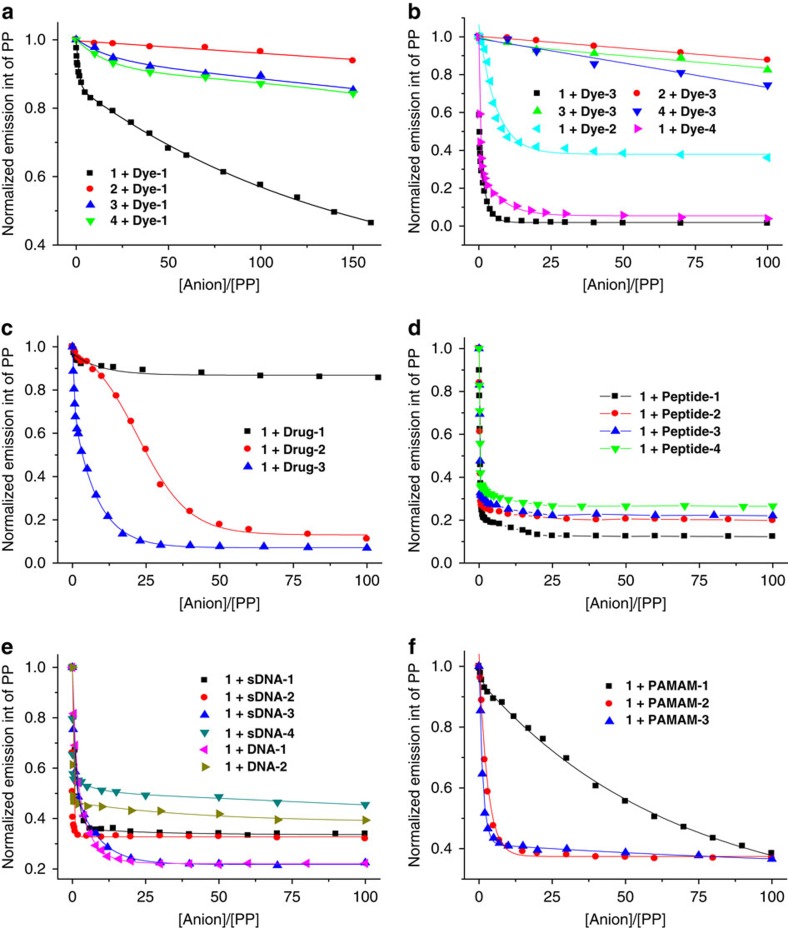
Solution-phase fluorescence quenching experiments. Normalized fluorescence quenching of the PP unit (0.02 mM) of **1**–**4** at 450 nm in water containing CB[8] (0.01 mM). (**a**) **1**–**4** by **Dye-1**. (**b**) **1**–**4** by **Dye-2**~**4**. (**c**) **1** by **Drug-1**~**3**. (**d**) **1** by **Peptide-1**~**4**. (**e**) **1** by **sDNA-1**~**4** and **DNA-1**,**2**. (**f**) **1** by **PAMAM-1**~**3**. [anion] represents the total concentration of the anionic unit of the guests.

It can be seen that all the 20 organic anions quenched the fluorescence of **1** much more efficiently than that of **2**, **3** or **4** (Fig. 6a,b). For the 3D SOF solution of **Com-Tetra**, all the guests, with the exception of **Dye-1** and **PAMAM-1**, could reach quenching saturation—a steady state when no more fluorescence change was observed upon addition of more guests (Fig. 6b–f). However, similar quenching saturation could not be realized by any of the guests tested for **Com-M**, **Com-D** and **Com-Tri**. Although quenching efficiency increased progressively from **Com-M** to **Com-D** to **Com-Tri**, which suggested multivalent interaction between the anionic guests and the cationic PP units of **Com-D** and **Com-Tri**, the much higher quenching efficiency for **Com-Tetra** relative to **Com-Tri** clearly suggests that **Com-Tetra** had an even stronger affinity towards guests, which may be attributed to the formation of the 3D framework that remarkably increased the apparent concentration of the PP unit in its interior and thus the overall electrostatic interaction. In the absence of CB[8], the same guest quenched the fluorescence of the PP unit of tetratopic **1** and tritopic **4** to a comparable extent, which further supported that it was the porous framework in **Com-Tetra** that caused the remarkable increase of the quenching efficiency. DLS experiments showed that after adding the guests, the *D*_H_ of **Com-Tetra** remained similar, which supported the high stability of the framework (Supplementary Fig. 25). It is noteworthy that all the quenching processes were quick and reached equilibrium within 1 min, which was needed for preparing the mixture solution and recording the spectrum, implying that even large nano-sized guests, such as **DNA-2** and **PAMAM-3**, could get into the interior of the 3D framework very quickly.

To collect more evidence for the inclusion of the guests in the interior of the porous framework in solution, we further performed DOSY experiments for **Dye-1**, **Drug-2** and **PAMAM-3**. Their diffusion coefficient (*D*) was determined to be 5.0 × 10^−10^, 3.9 × 10^−10^ and 3.2 × 10^−10^ m^2^ s^−1^ (Supplementary Figs 15–17), respectively. The values were all significantly larger than that (4.8 × 10^−11^ m^2^ s^−1^) of **Com-Tetra**. When the guests were added to the aqueous solution of **Com-Tetra**, the DOSY experiments showed that the signals of all the three components gave rise to a comparable *D* value (Supplementary Figs 18–20), which was actually identical to that of **Com-Tetra**. This result clearly indicated that the guest molecules were included into the interior of the 3D SOF and the framework of the SOF was still maintained after inclusion.

The above fluorescent and DOSY experiments also suggested that the new periodic framework was homogeneous. DOSY experiments gave rise to the identical result for the three samples after standing for 1 week, and centrifugation (8,000 r.p.m. for 1 h) for **COM-Tetra** and its mixture solution with **Dye-1**, **Drug-2** and **PAMAM-1** did not lead to any observable precipitate. These observations further supported the homogeneous nature of the periodic framework.

**Figure 7:**
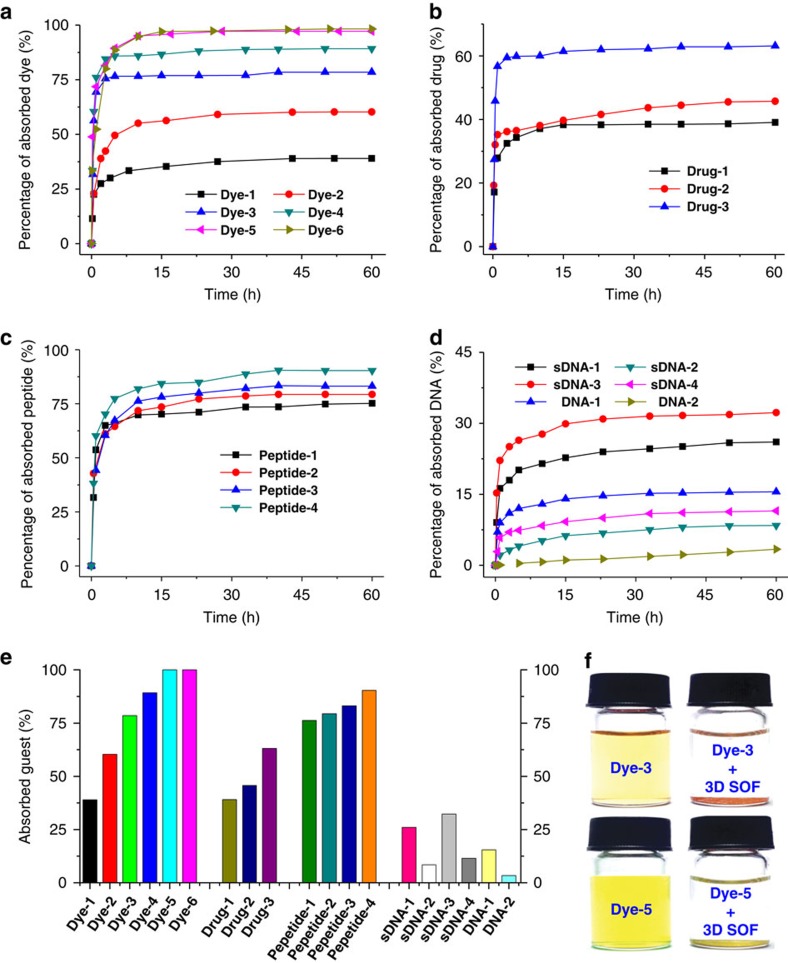
Absorption studies. Percentage of guests absorbed from water by microcrystals (1.0 mg) against time and sample pictures. (**a**) **Dye-1**~**6**, (**b**) **Drug-1**~**3**, (**c**) **Peptide-1**~**4**, (**d**) **sDNA-1**~**4** and **DNA-1**,**2**. (**e**) Percentage of guests absorbed by microcrystals after 60 h when the absorption reached equilibrium, except **DNA-2**. The initial anion concentration of all the guests was equal to [PP] of **1** of the microcrystals. The percentage was obtained from ultraviolet–vis absorption experiments. (**f**) The solution of **Dye-3** and **Dye-5** in water (left) and the identical solution after 60 h in the presence of microcrystals (1.0 mg) (right).

The microcrystals of **Com-Tetra**, once formed, became insoluble in water even at the boiling temperature. At temperatures above 120 °C, the microcrystals slowly dissolved in water in a sealed pressure bottle. As expected, after evaporation of the solvent, microcrystals could be formed again after standing for days, which clearly reflected the reversible feature of the self-assembling process. The high stability of the microcrystals in water at room temperature made it possible to quantitatively evaluate their capacity of extracting conjugated anionic guests from their aqueous solutions using ultraviolet–visible (vis) spectroscopy. Similar to the solution behaviour, **Com-Tetra** functioned like an ‘ion sponge’ that displayed differential binding affinity towards a wide variety of organic anions. In a typical experiment, microcrystals (1.0 mg, 0.24 μmol) were immersed in an aqueous solution (1.0 ml) of a guest compound whose anion concentration was made equal to the concentration of the PP unit of **1** in the microcrystals. The quantity of the guest in solution was measured using ultraviolet–vis spectroscopy by monitoring its characteristic absorption over a period of about 60 h, from which the amount of the guest extracted by the 3D SOF was calculated. The results are provided in Fig. 7. It can be found that the extraction reached equilibrium for all the guests within 60 h except **DNA-2**, extraction for which was much slower. We tentatively attribute this exception to the large size of DNA, which has a diameter of about 2.0 nm and cannot easily pass the more rigid aperture of the SOF microcrystal^48^. This result also implied that in solution the homogeneous self-assembled SOF were elastic, and could be expanded to allow for quick inclusion of **DNA-2**, as evidenced by fast fluorescence quenching, and evaporation of the solvent might lead to its shrinking in the solid state. Remarkably, the absorption percentage of eight guests exceeded 75%, and for trianion **Dye-5** and tetraanion **Dye-6**, the adsorption was nearly quantitative (>99%). These values may be lower limits because some of the SOF complex may have remained in solution, which should also absorb a little guest that could be detected. The X-ray diffraction profile of the microcrystals that quantitatively absorbed **Dye-5** displayed a diffraction pattern similar to that of dye-free microcrystals (Supplementary Fig. 26), indicating that the periodic structure was maintained after guest absorption. This result is also consistent with the above solution-phase DLS experiments (Supplementary Fig. 25), supporting the high stability of the supramolecular framework in the microcrystals.

### Release studies

**Figure 8:**
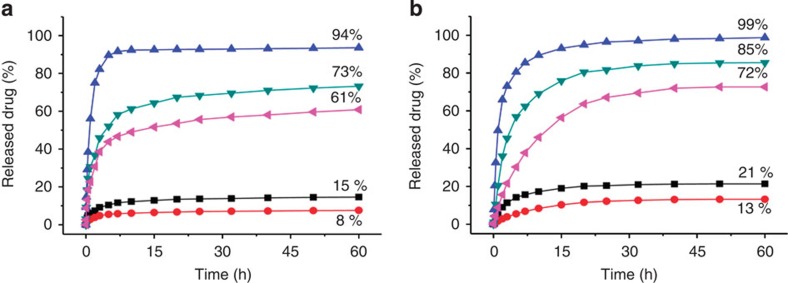
Release of microcrystal (1 mg)-adsorbed drugs at 37 °C against time. (**a**) In 1.0 ml of water containing 1.0 equiv of hydrochloric acid relative to the molar amount of the Na^+^ ion of the attracted guests. (**b**) In 1.0 ml of CH_3_CO_2_H/CH_3_CO_2_Na (pH=4.5) buffer. The experiments were conducted with the samples being in the static state (blue triangles: **Drug-3**, black squares: **Drug-1** and red circles: **Drug-2**) or on a shaking orbital shaker (green triangles: **Drug-1** and purple triangles: **Drug-2**).

The release properties of the drug-loaded microcrystals were further evaluated by exposing the isolated microcrystals with pre-adsorbed **Drugs 1~3** in acidic media. For the first series of experiments, the microcrystals were immerged in 1 ml of water at 37 °C and then 1.0 equiv of hydrochloric acid relative to the molar amount of the Na^+^ ion of the included guests was added. The pH was about 6.5–6.8. The release profiles were determined by following the solution absorption of the drugs at different times (Fig. 8a). It can be seen that, when the release proceeded with the samples being static, after 60 h, the drug release contents were 15, 8 and 94% for **Drug-1, Drug-2** and **Drug-3**, respectively. When the release occurred on a shaking orbital shaker, the release contents of **Drug-1** and **Drug-2** could be increased to 73 and 61%, respectively. The acid-responsive drug release was reproduced in a slightly different aqueous media when the solution pH was kept at 4.5 using a CH_3_CO_2_H/CH_3_CO_2_Na buffer (Fig. 8b)^49^. When the samples were kept static, the release content for **Drug-3** was 99% after 60 h. When the release was conducted on a shaking orbital shaker, the release contents of **Drug-1** and **Drug-2** were increased from 21 and 13% to 85 and 72%, respectively.

### Cytotoxicity test

**Figure 9:**
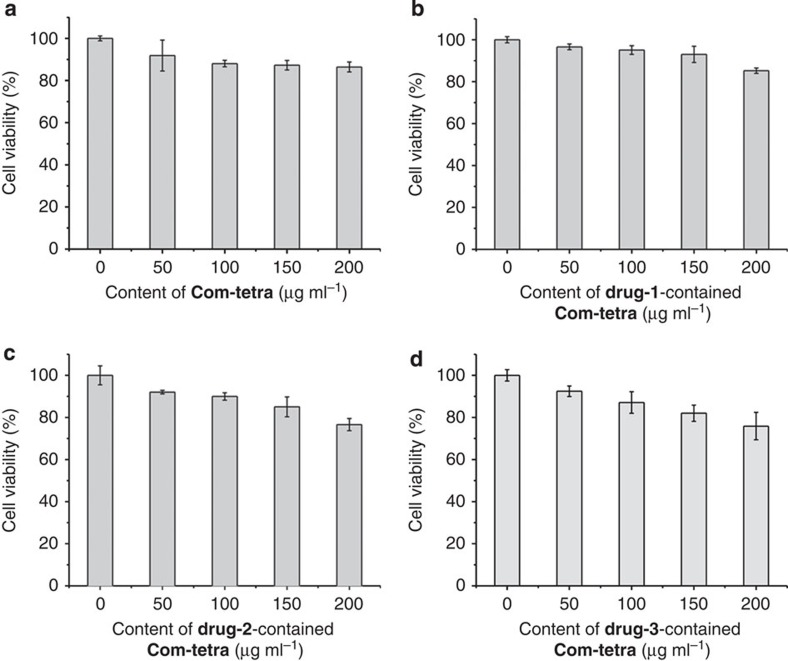
Cytotoxicity of Com-Tetra. Cell viability values (%) estimated by CCK-8 proliferation tests versus incubation concentrations microcrystals of (**a**) **Com-Tetra**. (**b**) **Drug-1**-contained **Com-Tetra**. (**c**) **Drug-2**-contained **Com-Tetra**. (**d**) **Drug-3**-contained **Com-Tetra**. HeLa cells (~2 × 10^4^ per well) were incubated with 50–200 μg ml^−1^ samples at 37 °C for 24 h. Error bars represent the s.d. of uncertainty for each point.

Human cancer (HeLa) cells were used as the model to evaluate the *in vitro* cytotoxicity of **Com-Tetra** and the drug-contained **Com-Tetra** samples using the Cell Counting Kit-8 (CCK-8) assay. The results are provided in Fig. 9. It can be seen that the viability of the HeLa cells maintained at 91.8–96.6%, 87.1–95.1% or 75.9–86.4% after incubating for 24 h with the samples at 50, 100 and 200 μg ml^−1^, respectively. These preliminary results show that the cytotoxicity of the new 3D SOF is low^50^.

### Gas adsorption

Nitrogen and carbon dioxide gas absorption capabilities of the microcrystals were also evaluated at −196 and 0 °C, respectively, after the microcrystals were activated under vacuum at 180 °C for 10 h (Supplementary Figs 41 and 42). The nitrogen gas absorption amount was 5.4 cm^3^ (STP) g^−1^ at *P/P*_0_=1.0, and the Brunauer-Emmett-Teller (BET) surface area of the microcrystals was 10.3 m^2^ g^−1^. The carbon dioxide gas absorption amount was 0.28 mmol g^−1^ at 800 mm Hg. The microcrystals after the activation still maintained the structural ordering because their X-ray diffraction profile was nearly the same as that of the sample before activation. These preliminary results show that the new supramolecular microcrystals are not good porous materials for gas absorption.

## Discussion

We have successfully constructed the first periodic 3D SOF in solution using a robust self-assembling strategy in water. By virtue of the rigidity of the tetrahedral building block and the high stability of hydrophobically driven encapsulation that holds the building blocks together, the resulting periodic framework possesses high porosity that allows for inclusion of a variety of organic and biologically active guests into its interior. The solution-phase 3D framework represents a new type of ordered supramolecular polymer that resembles crystalline metal–organic frameworks and covalent organic frameworks in periodicity and porosity, but differs greatly by much enhanced reversibility and solution processability. It also provides new opportunities for the design of novel soft porous architectures. For example, stable ordered 3D polymers might be constructed after covalently locking the framework. Importantly, such a 3D framework can maintain the periodicity and porosity in the solid state. The resulting microcrystals are able to extract various guests from their solutions—in some cases even reaching a charge-stoichiometric equilibrium—and release the included guests in a controllable manner. Such guest inclusion and release properties at the heterogeneous solid–liquid mixed phases also make the 3D SOF solid a great candidate for material applications such as membranes, sensors and controlled delivery.

## Methods

### Materials and measurements

All reagents were obtained from commercial suppliers and used without further purification unless otherwise noted. All reactions were carried out under a dry nitrogen atmosphere. All solvents were dried before use following standard procedures. Column chromatography was performed on silica gel (200–300 mesh), and thin-layer chromatography was performed on precoated silica gel plates (0.4–0.5 mm thick). ^1^H and ^13^C NMR spectra were recorded with a 400-MHz spectrometer in the indicated solvents at 25 °C (Supplementary Figs 30–37). Variable-temperature ^1^H NMR spectra were recorded with a 500-MHz spectrometer in the indicated solvents. ^1^H NMR DOSY experiments were carried out with a 400 NMR spectrometer. Chemical shifts were referenced to the residual solvent peaks. Mass spectra (electrospray ionization) were obtained on Shimadzu LCMS-2010EV, IonSpec 4.7 Tesla FTMS and microTOF II spectrometers. DLS experiments were performed on a Malvern Zetasizer Nano ZS90 light-scattering Instrument. Powder X-ray diffraction measurements were carried out on a Bruker D8 Advance diffractometer at 40 kV and 40 mA with Cu Kα radiation (*λ*=1.5406 Å). Scanning electron micrographs and elemental distribution of the samples were obtained on a JSM-6330F Field Emission Scanning Electron Microscope combined with energy dispersive X-ray analysis. Transmission electron micrographs were recorded on a 2100F JEOL FETEM microscope at 120 kv aligned for low dose (10 e Å^−2^ s^−1^) diffractive imaging. The synthesis and characterization, and spectroscopic measurements, including DLS, fluorescence, ultraviolet–vis absorption, thermogravimetric analysis, gas adsorption experiments and cytotoxicity experiments are summarized in the Supplementary Methods.

### Solution-phase synchrotron X-ray diffraction data collection

The X-ray diffraction data were obtained at beamline BL14B1 of the Shanghai Synchrotron Radiation Facility at a wavelength of 1.2398 Å. BL14B1 is a beamline based on a bending magnet and a Si (111) double crystal monochromator was employed to monochromatize the beam. The size of the focus spot is about 0.5 mm and the end station is equipped with a Huber 5021 diffractometer. NaI scintillation detector was used for data collection.

### Solid-phase synchrotron X-ray-scattering data collection

Synchrotron radiation SAXS experiments were performed on the BL16B beamline of Shanghai Synchrotron Radiation Facility, using a fixed wavelength of 0.124 nm, a sample-to-detector distance of 2.01 m and an exposure time of 2,000 s. The 2D scattering pattern was collected on a charge-coupled device camera, and the curve intensities versus *q* were obtained by integrating the data from the pattern.

### Solution-phase synchrotron SAXS data collection

The SAXS data were collected at the SIBYLS Beamline 12.3.1 of the Advanced Light Source (Lawrence Berkeley National Laboratory). The **Com-Tetra** ([PP]=8 mM) complex in water were exposed for 0.5 and 1, and 6 s, followed by a 1-s exposure to check for radiation damage, using a MARCCD X-ray detector system located 1.6 m from the sample chamber to collect data in the *q*-spacing 0.01–0.32 Å^−1^, where *q*=4*π*sin*θ*/*λ* (2*θ* is the scattering angle and *λ* is the wavelength (1.03 Å)). Filtrates resulting from the sample concentration process were exposed for equivalent times and the intensities were subtracted from the corresponding sample exposures. Data from the low- and high-resolution ranges of the respective short and long exposures were scaled and merged to obtain the final data sets shown in Fig. 3a.

## Author contributions

Z.-T.L., D.-W.Z. and Y.L. conceived and designed the research, analysed the data and composed the manuscript. J.T. carried out most of the experiments, analysed the data and composed the manuscript. T.-Y.Z., S.-C.Z., S.A., M.V.A. and S.-H.X. carried out part of the experiments. H.W. and X.Z. analysed the data. All authors discussed and commented on the manuscript.

## Additional information

**How to cite this article:** Tian, J. *et al.* Three-dimensional periodic supramolecular organic framework ion sponge in water and microcrystals. *Nat. Commun.* 5:5574 doi: 10.1038/ncomms6574 (2014).

## Supplementary Material

Supplementary Information Supplementary Figures 1-42, Supplementary Methods and Supplementary References
