# Supplementary material for: Three-dimensional periodic supramolecular organic framework ion sponge in water and microcrystals
Source: Nat Commun. 2014 Dec 3;5:5574. doi: 10.1038/ncomms6574 (PMC4268711; doi:10.1038/ncomms6574)
Supplement: Supplementary Information — Supplementary Figures 1-42, Supplementary Methods and Supplementary References [file ncomms6574-s1.pdf]

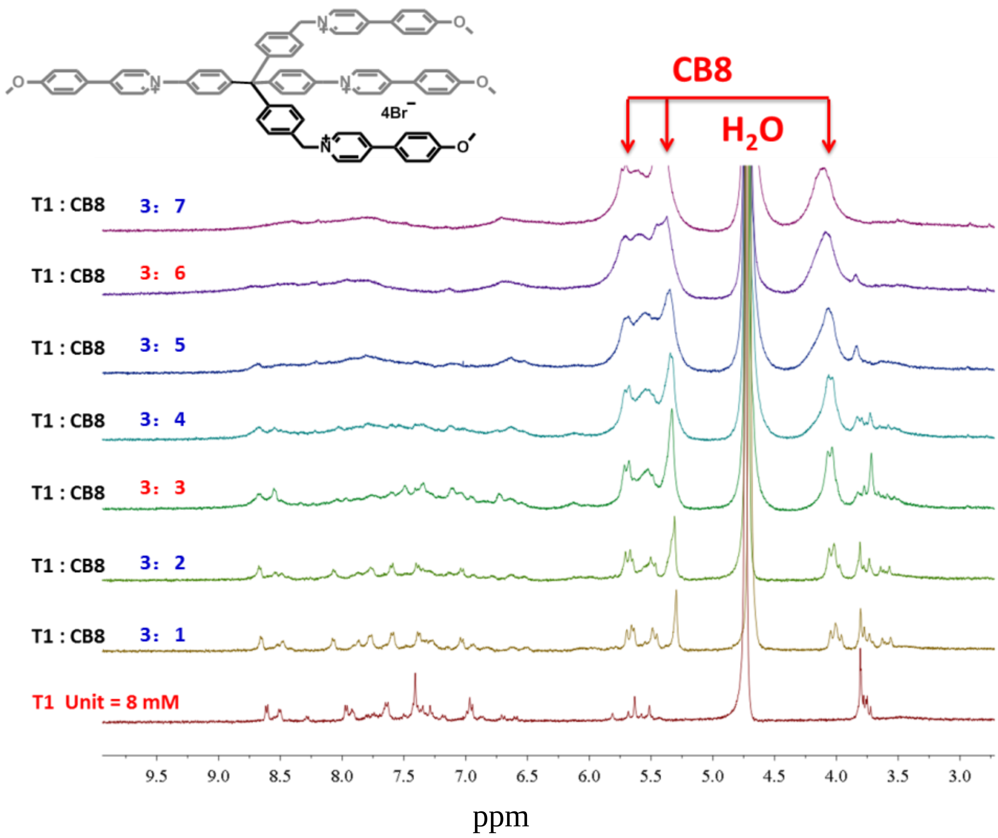

Supplementary Figure 1 | ^{1}H NMR spectra. Titration of **1** in D_2O with increasing amounts of CB[8] (400 MHz, 25 °C, [PP] = 2[CB[8]] = 8.0 mM).

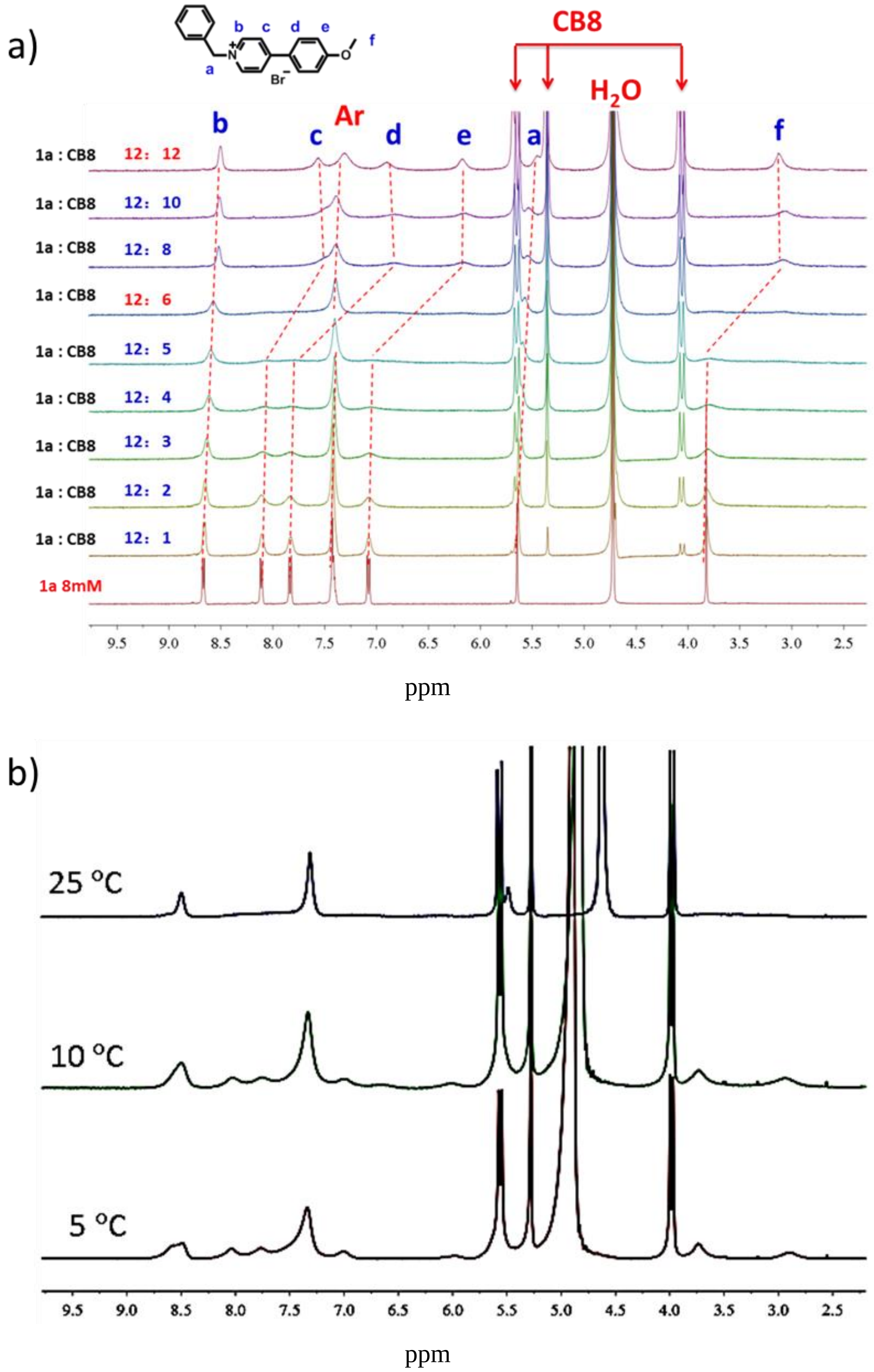

Supplementary Figure 2 | ^{1}H NMR spectra. (a) Titration of **2** in D_2O with increasing amounts of CB[8] (400 MHz, 25 °C, [PP] = 2[CB[8]] = 8.0 mM). (b) Variable-temperature spectrum (400 MHz) of the 2:1 solution of **2** (8.0 mM) and CB[8] in D_2O.

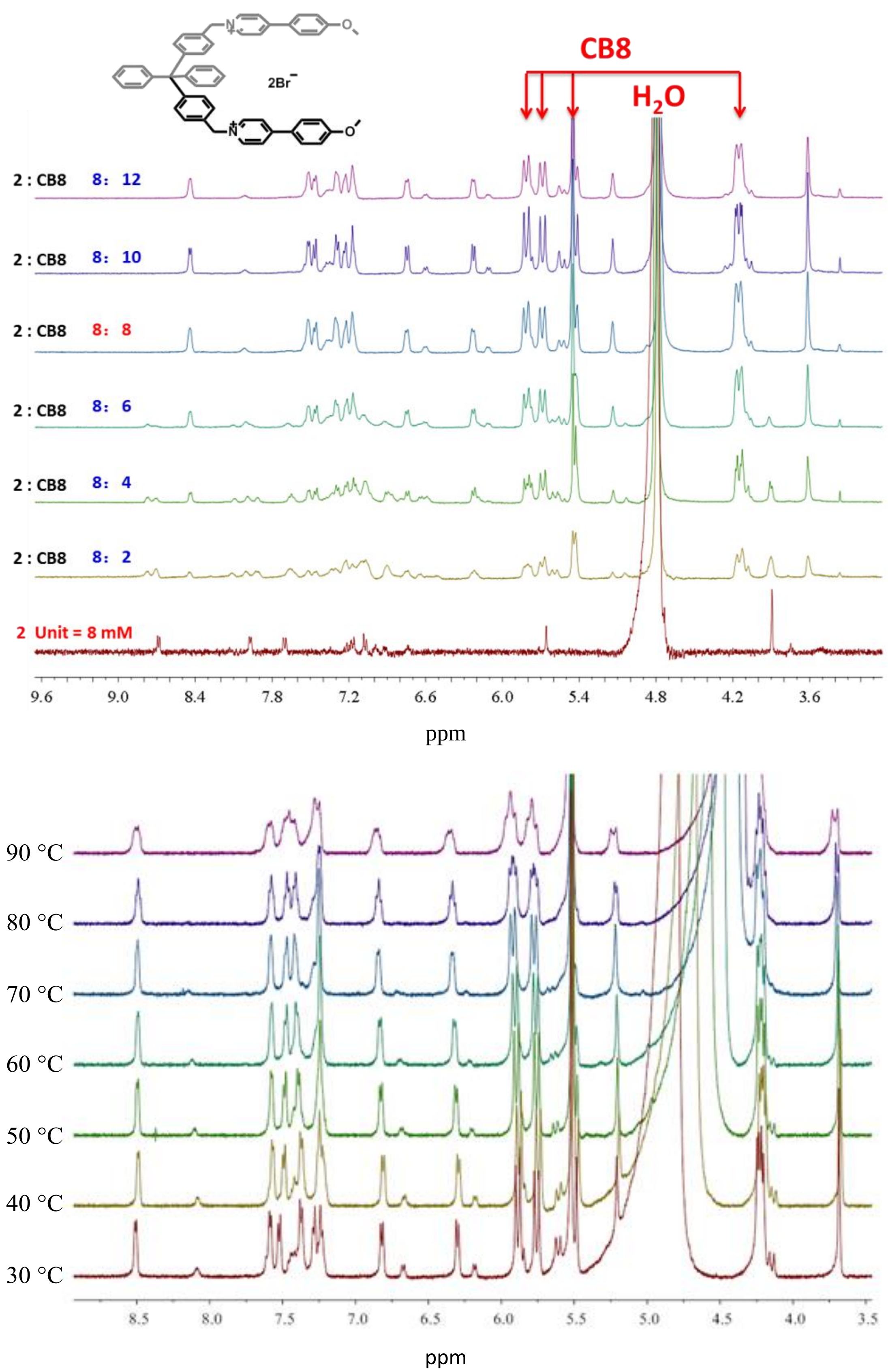

Supplementary Figure 3 | ^{1}H NMR spectra. Upper: Titration of **3** in D_2O with increasing amounts of CB[8] (400 MHz, 25 °C, [PP] = 2[CB[8]] = 8.0 mM). Down: Spectra at different temperatures (30-90 °C).

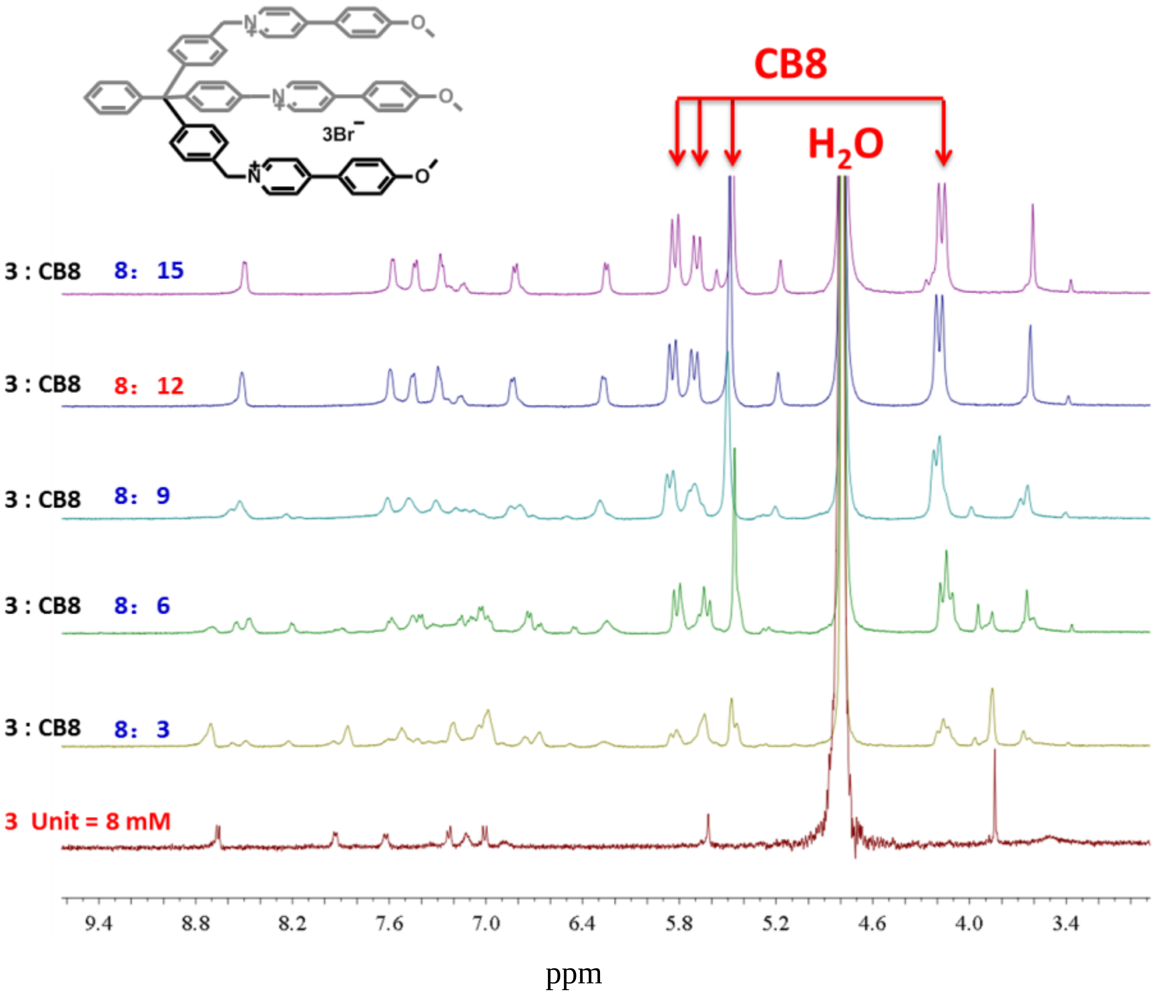

Supplementary Figure 4 | ^{1}H NMR spectra. Titration of **4** in D_2O with increasing amounts of CB[8] (400 MHz, 25 °C, [PP] = 2[CB[8]] = 8.0 mM).

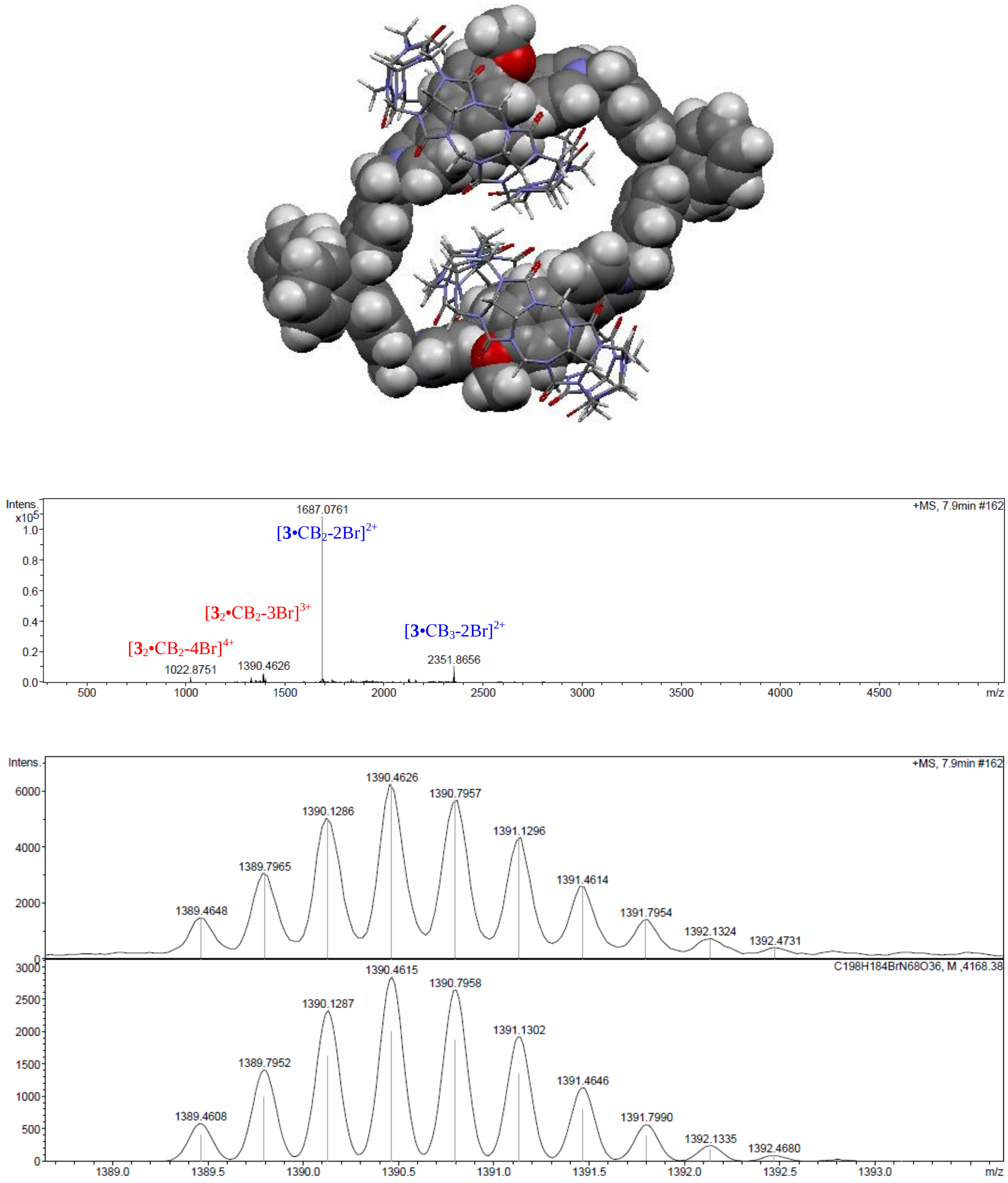

Supplementary Figure 5 | ESI-mass spectrum of the complex of **3** and CB[8] (1:1).

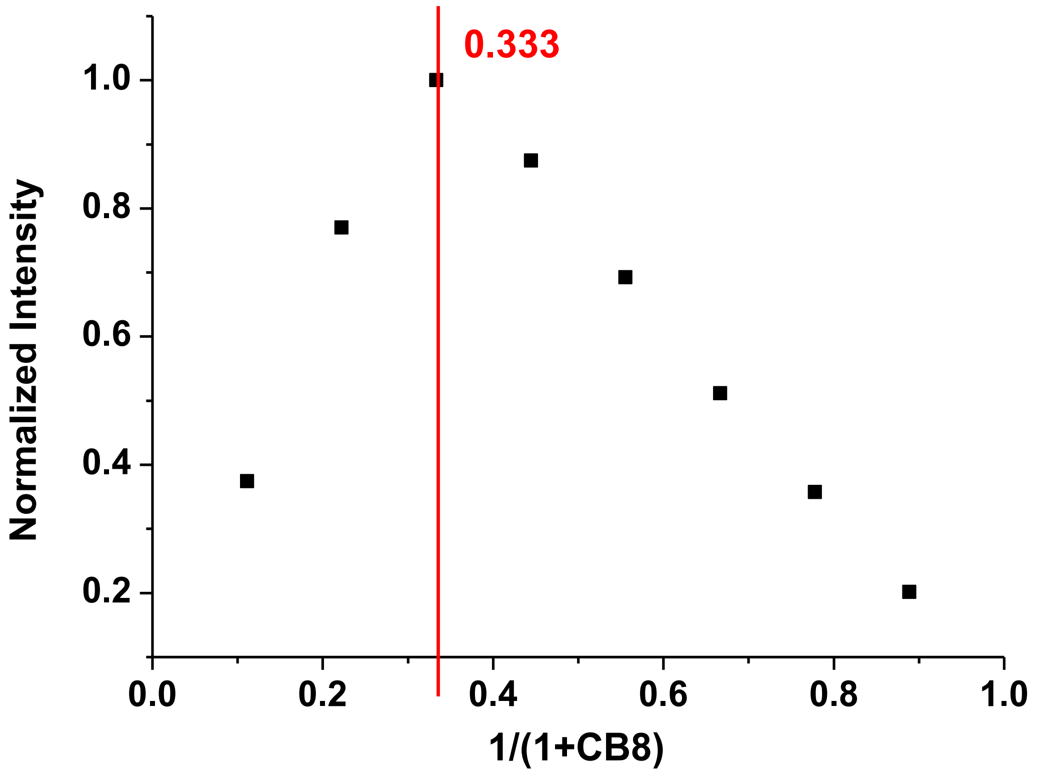

Supplementary Figure 6 | Job's plot. Plot obtained upon mixing **1** and CB[8]. Solvent: H_2O; T = 25 °C; $[\mathbf{1}]_{total}$ + $[CB[8]]_{total}$ = 1.5×10^{-4} M; ex/em = 400 nm/500 nm.

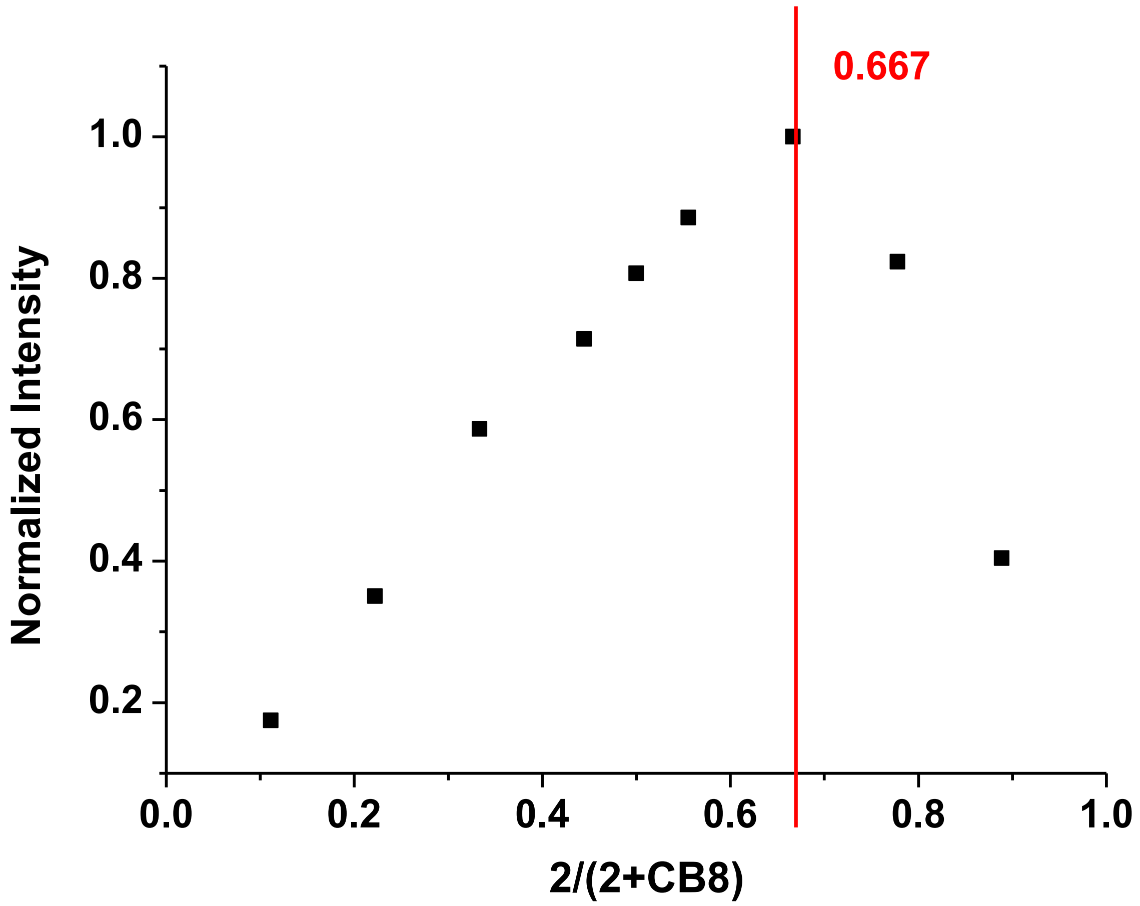

Supplementary Figure 7 | Job's plot. Plot obtained upon mixing **2** and CB[8]. Solvent: H_2O; T = 25 °C; [**2**]$_{total}$ + [CB[8]]$_{total}$ = 1.5×10^{-4} M; ex/em = 380 nm/450 nm.

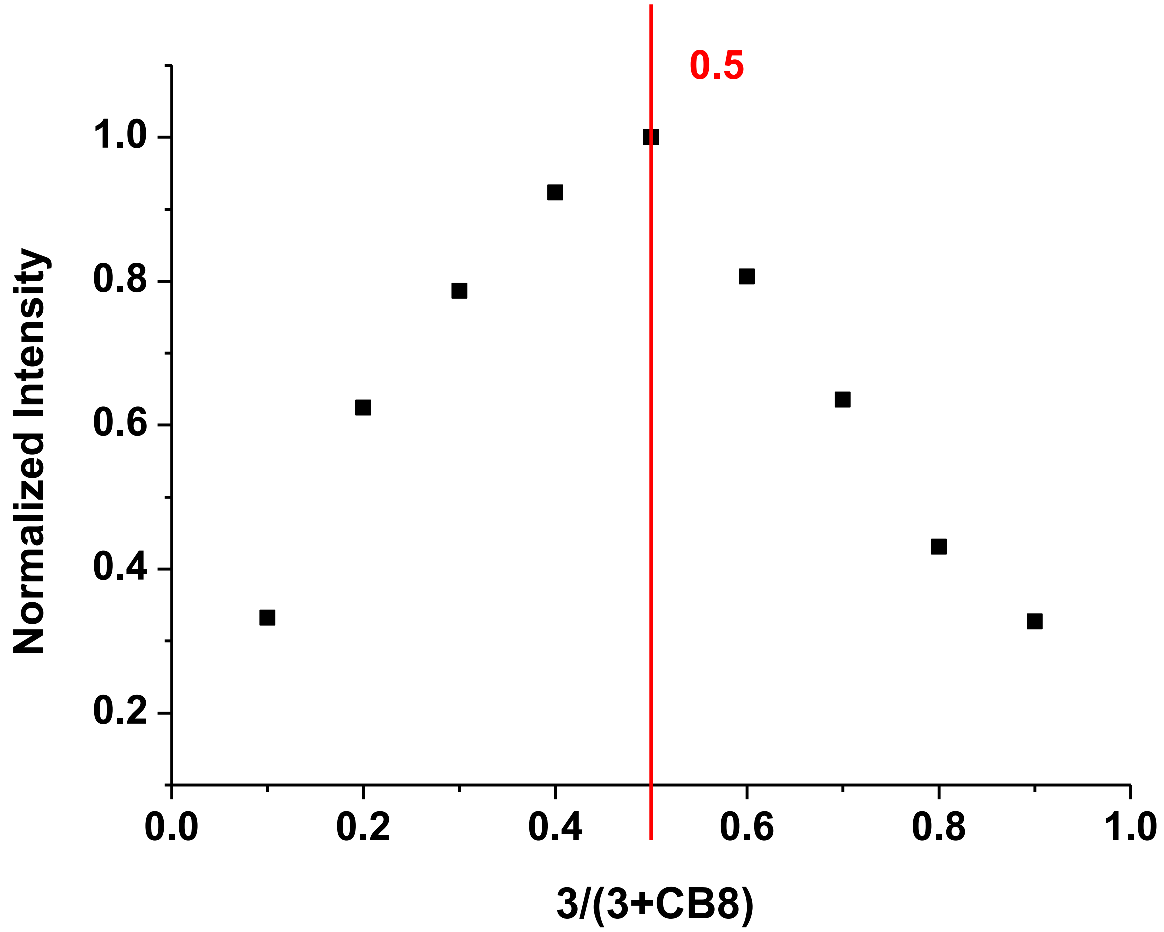

Supplementary Figure 8 | Job's plot. Plot obtained upon mixing **3** and CB[8]. Solvent: H_2O; T = 25 °C; [**3**]$_{total}$ + [CB[8]]$_{total}$ = 5×10^{-5} M; ex/em = 380 nm/500 nm.

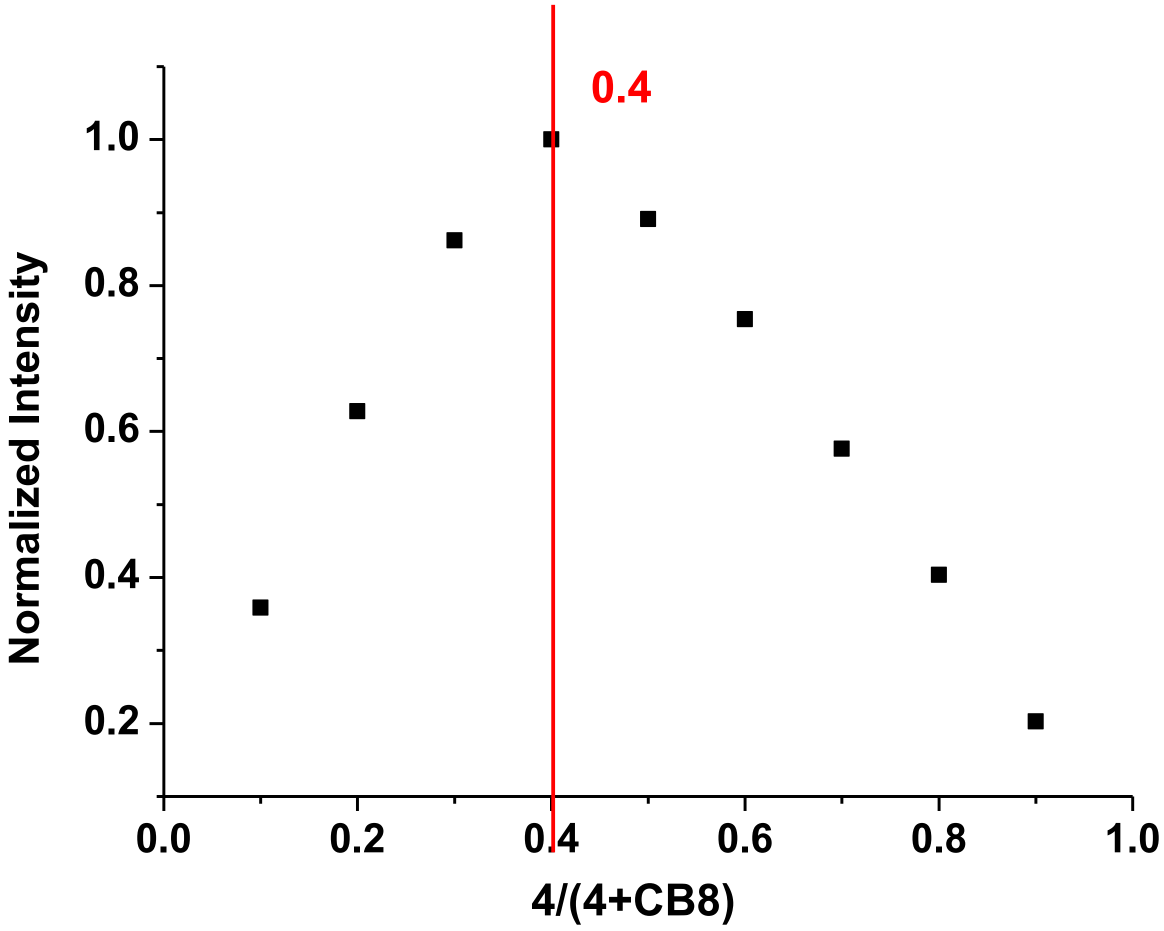

Supplementary Figure 9 | Job's plot. Plot obtained upon mixing **4** and CB[8]. Solvent: H_2O; T = 25 °C; $[\mathbf{4}]_{total}$ + $[CB[8]]_{total}$ = 5 × 10^{-5} M; ex/em = 380 nm/500 nm.

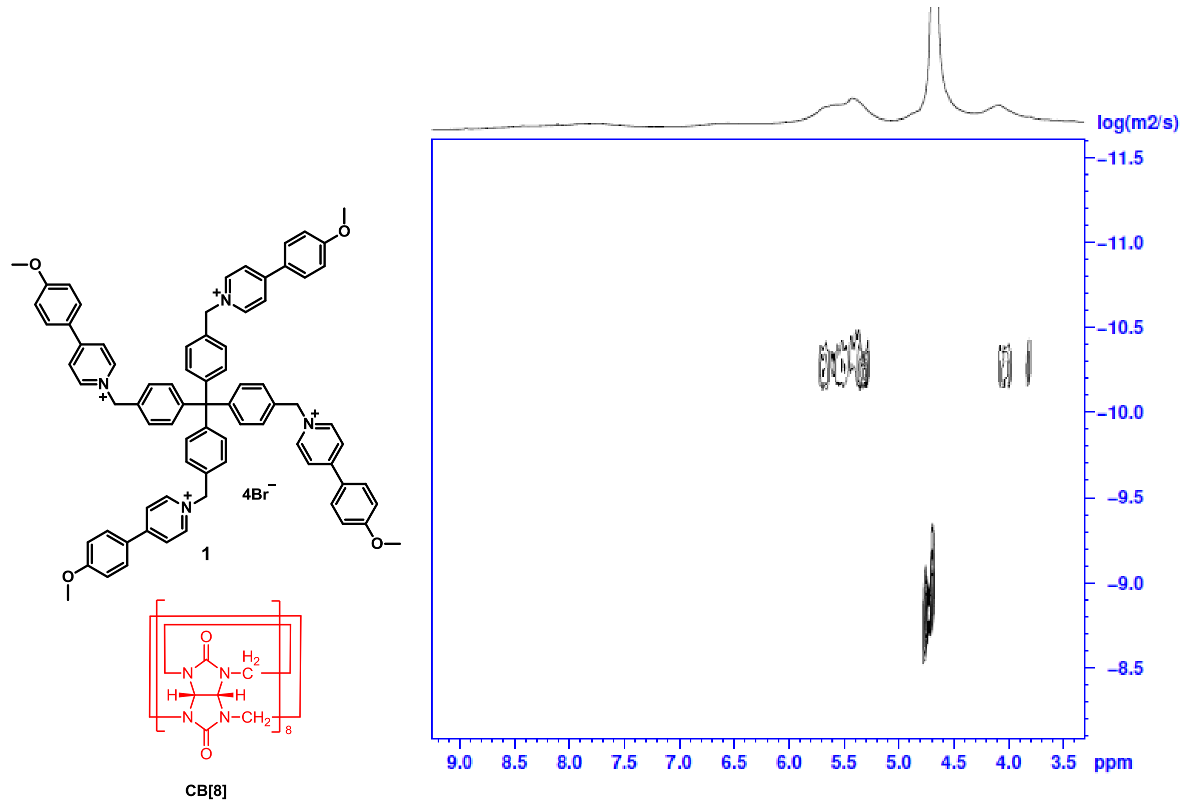

Supplementary Figure 10 | DOSY-^{1}H NMR spectrum (400 MHz). The solution of **1** (2 mM) and CB[8] (4 mM) in D_2O. The ordinate represents the log value of the diffusion constant.

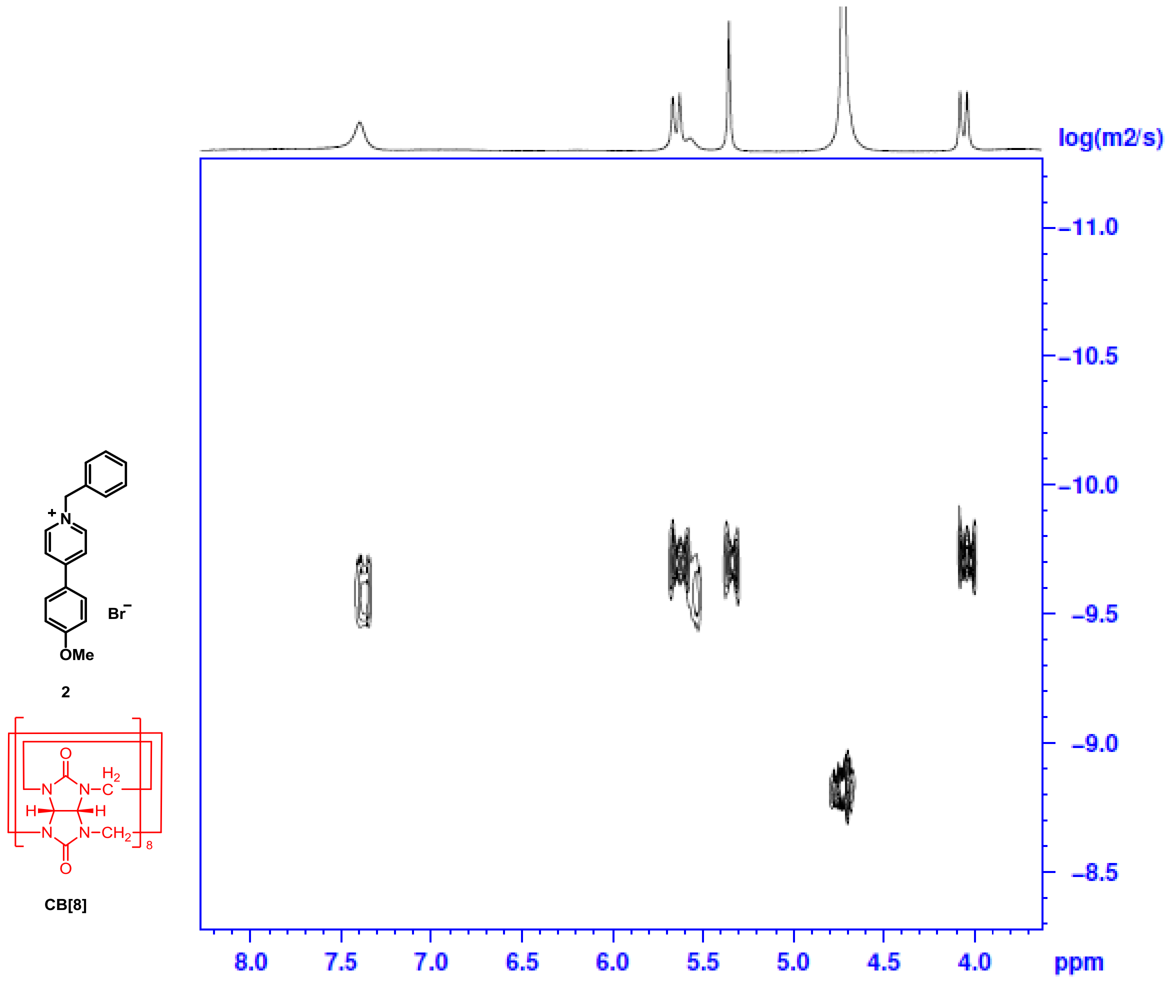

Supplementary Figure 11 | DOSY-^{1}H NMR spectrum (400 MHz). The solution of **2** (4 mM) and CB[8] (2 mM) in D_2O. The ordinate represents the log value of the diffusion constant.

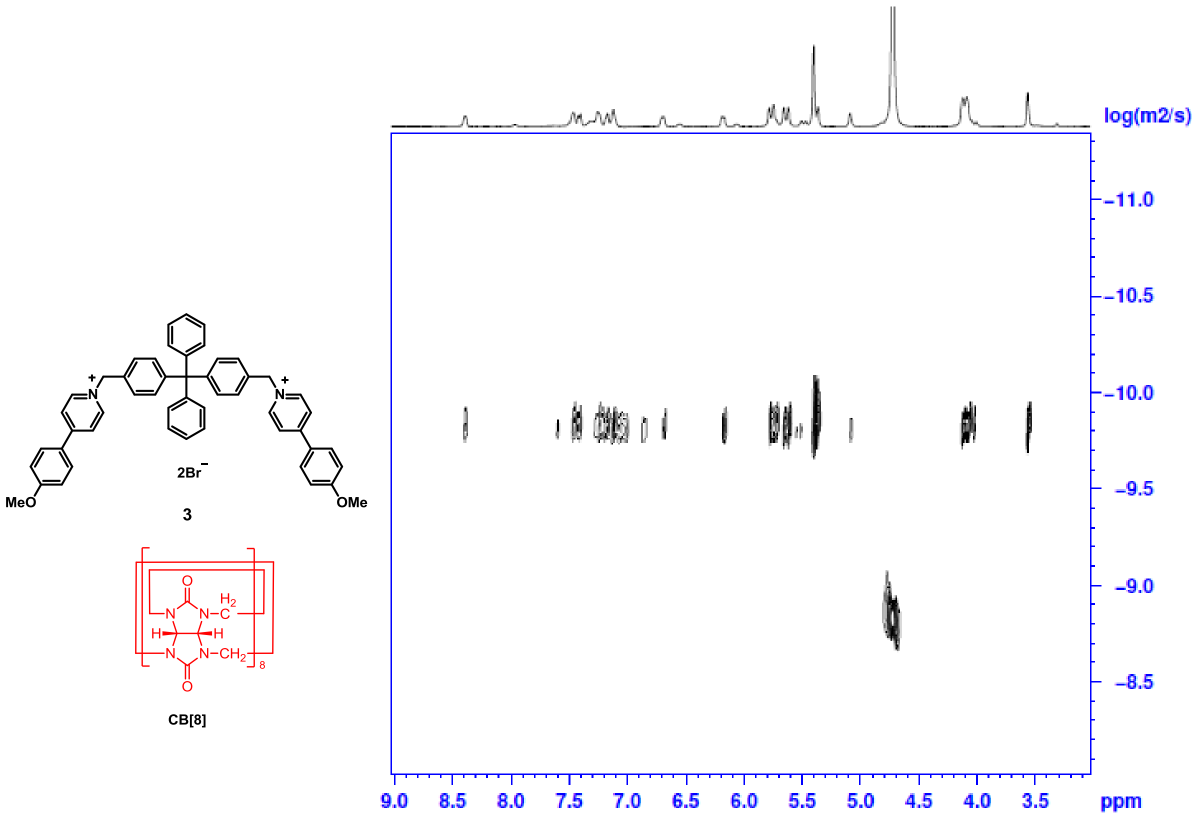

Supplementary Figure 12 | DOSY-^{1}H NMR spectrum (400 MHz). The solution of **3** (2 mM) and CB[8] (2 mM) in D_2O. The ordinate represents the log value of the diffusion constant.

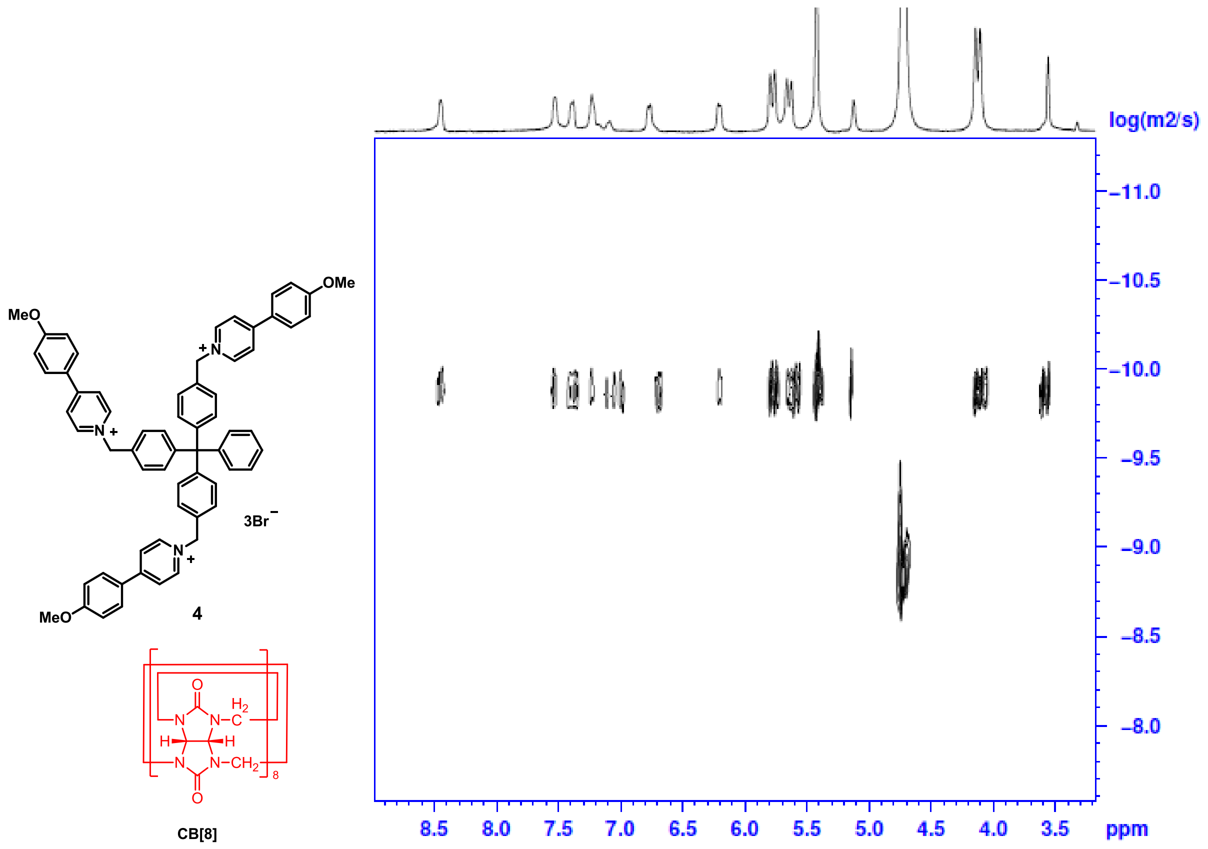

Supplementary Figure 13 | DOSY-^{1}H NMR spectrum (400 MHz). The solution of **4** (2 mM) and CB[8] (3 mM) in D_2O. The ordinate represents the log value of the diffusion constant.

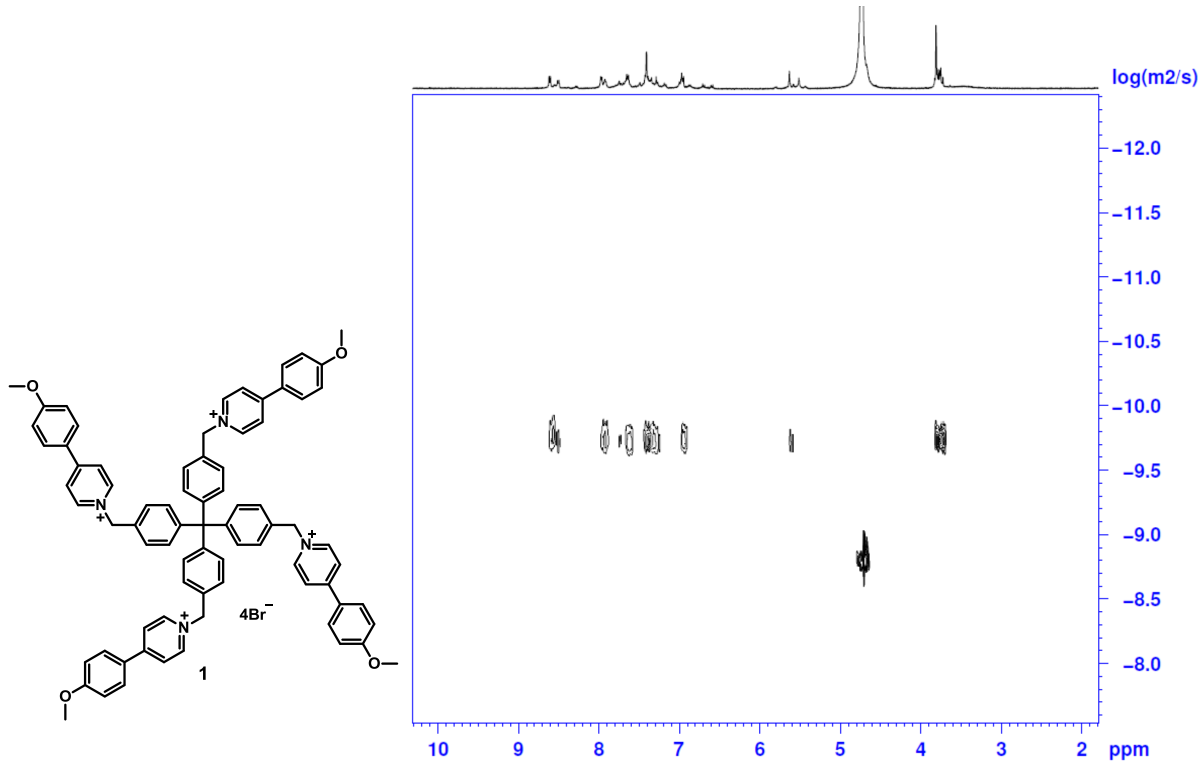

Supplementary Figure 14 | DOSY-^{1}H NMR spectrum (400 MHz). The solution of **1** (2 mM) in D_2O. The ordinate represents the log value of the diffusion constant.

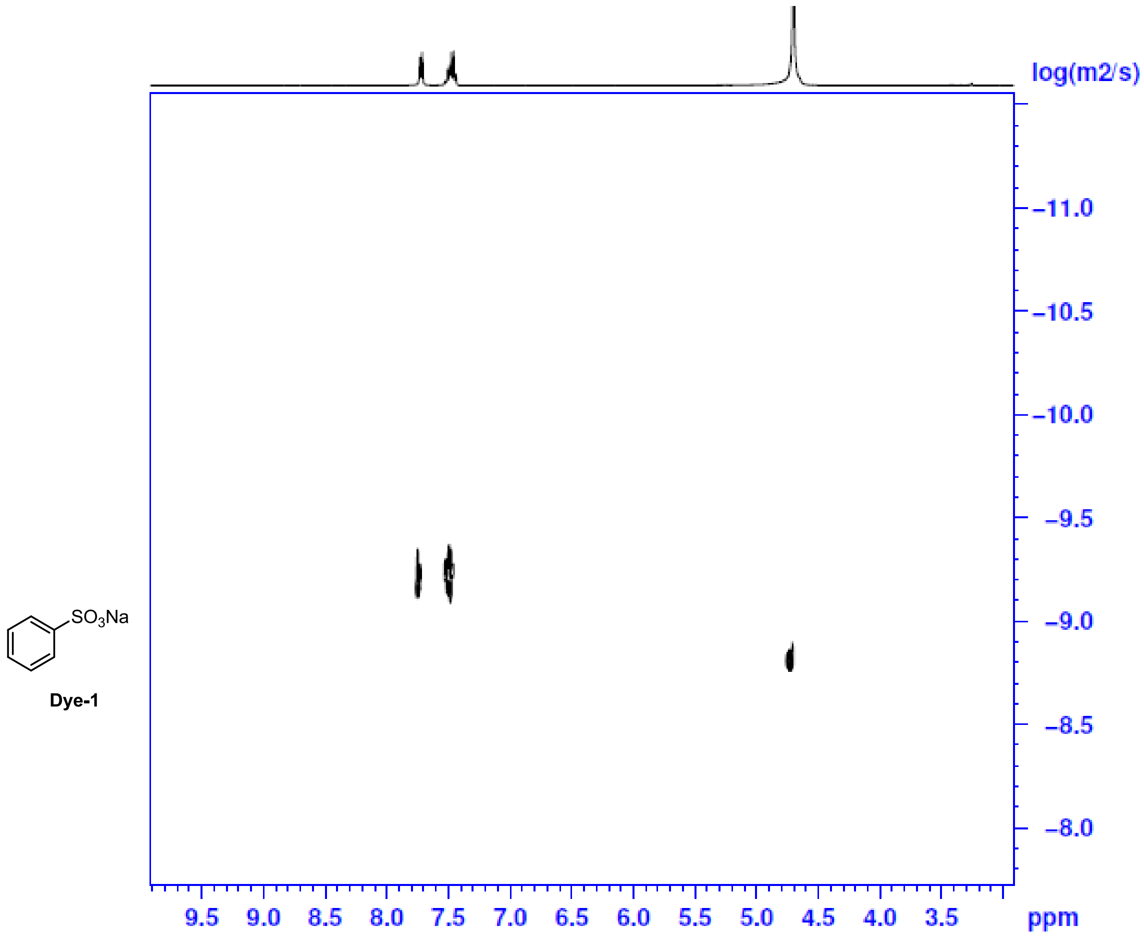

Supplementary Figure 15 | DOSY-^{1}H NMR spectrum (400 MHz). The solution of **Dye-1** (8 mM) in D_2O. The ordinate represents the log value of the diffusion constant.

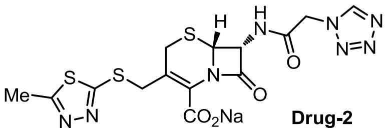

Cefazolin Sodium Salt

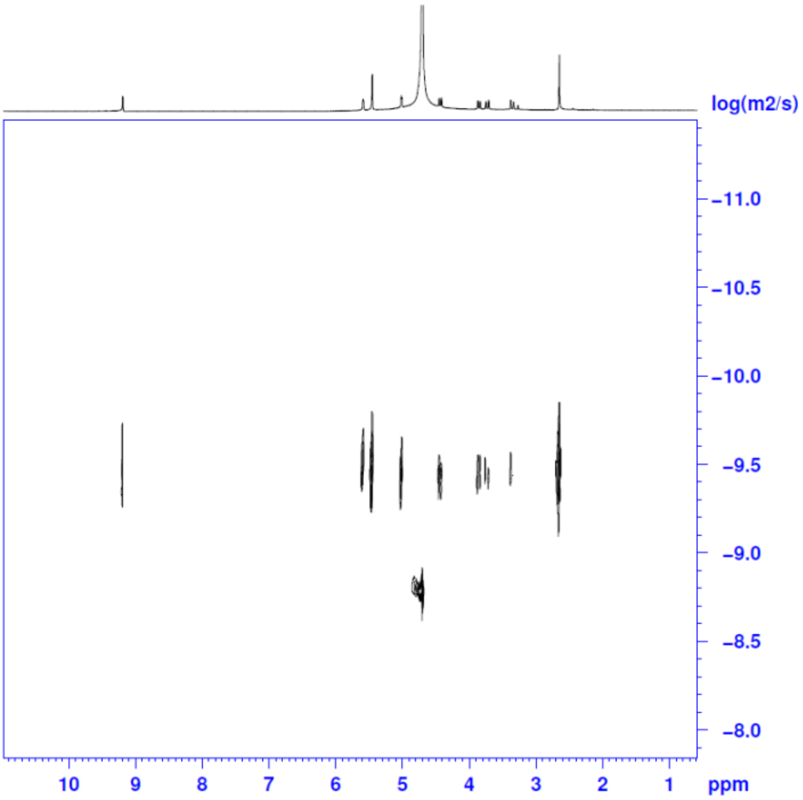

Supplementary Figure 16 | DOSY-^{1}H NMR spectrum (400 MHz). The solution of **Drug-2** (8 mM) in D_2O. The ordinate represents the log value of the diffusion constant.

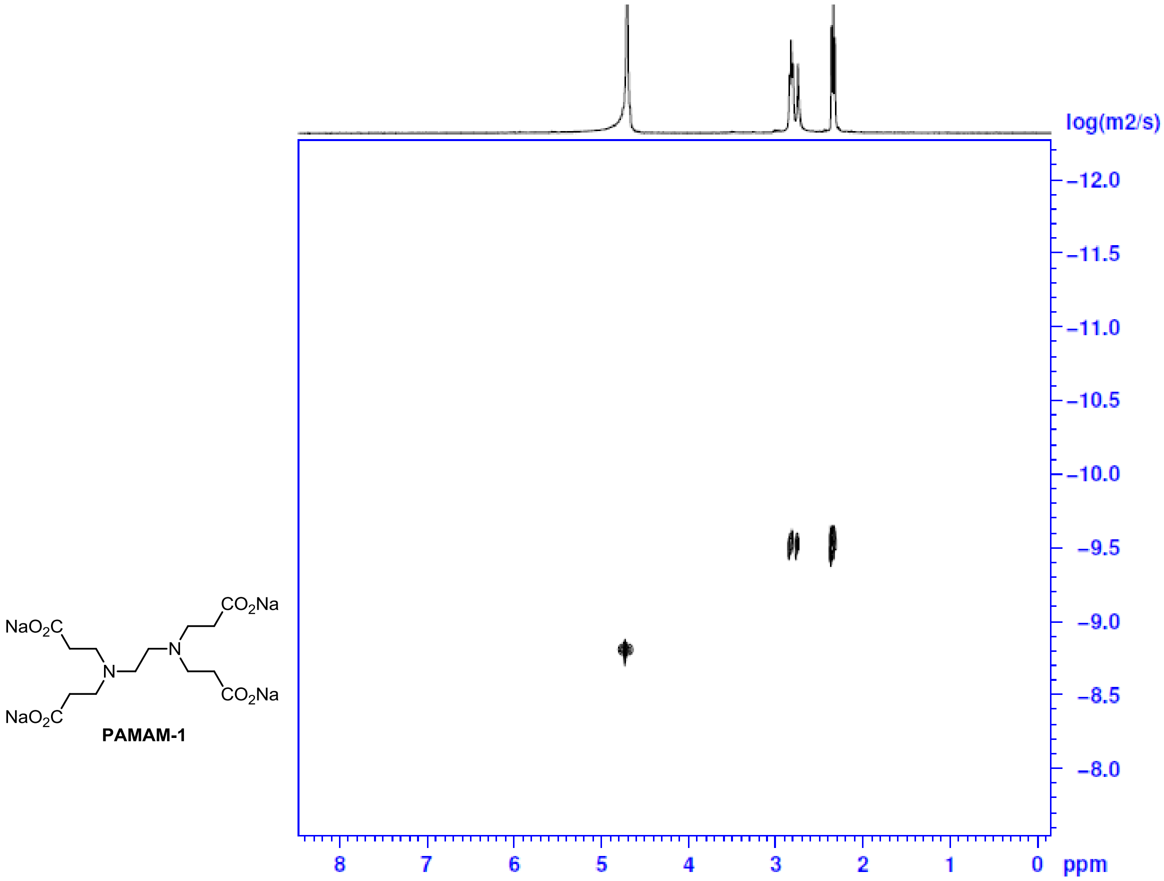

Supplementary Figure 17 | DOSY-^{1}H NMR spectrum (400 MHz). The solution of **PAMAM-1** (2 mM) in D_2O. The ordinate represents the log value of the diffusion constant.

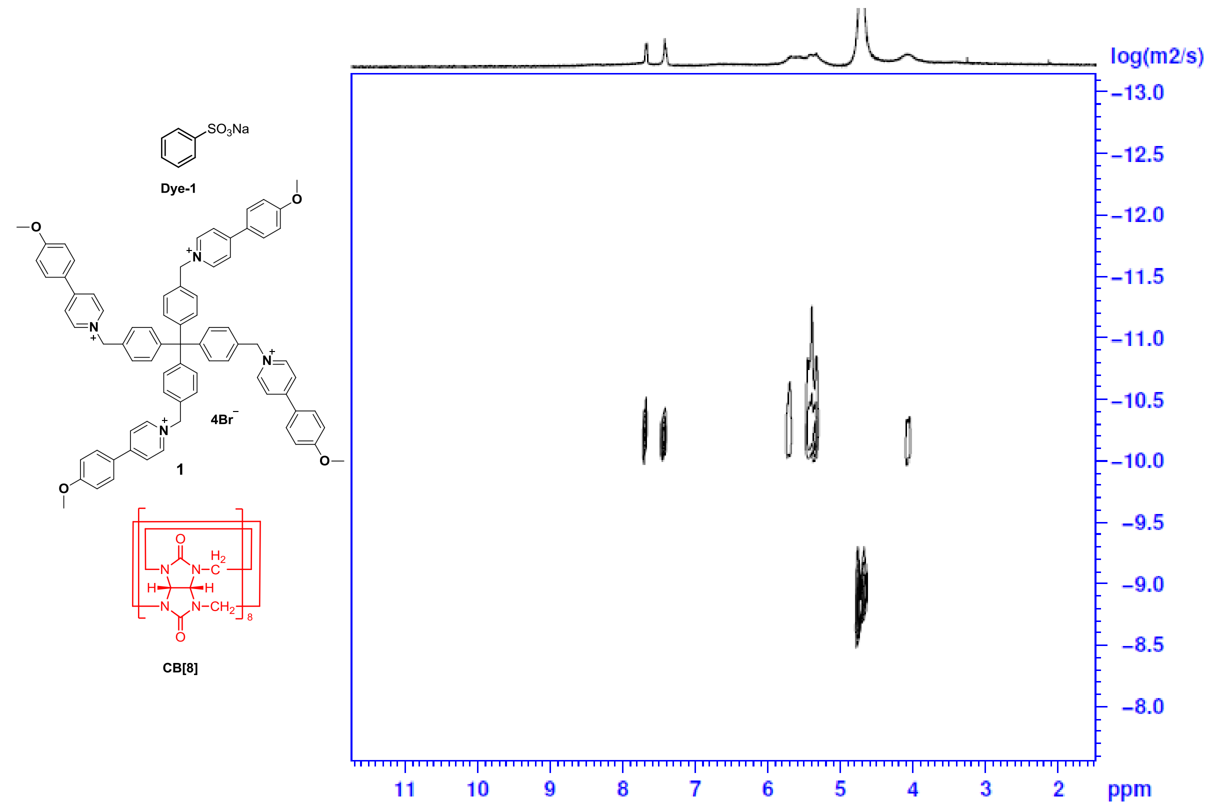

Supplementary Figure 18 | DOSY-^{1}H NMR spectrum (400 MHz). The solution of **1** (2 mM), CB[8] (4 mM) and **Dye-1** (8 mM) in D_2O. The ordinate represents the log value of the diffusion constant.

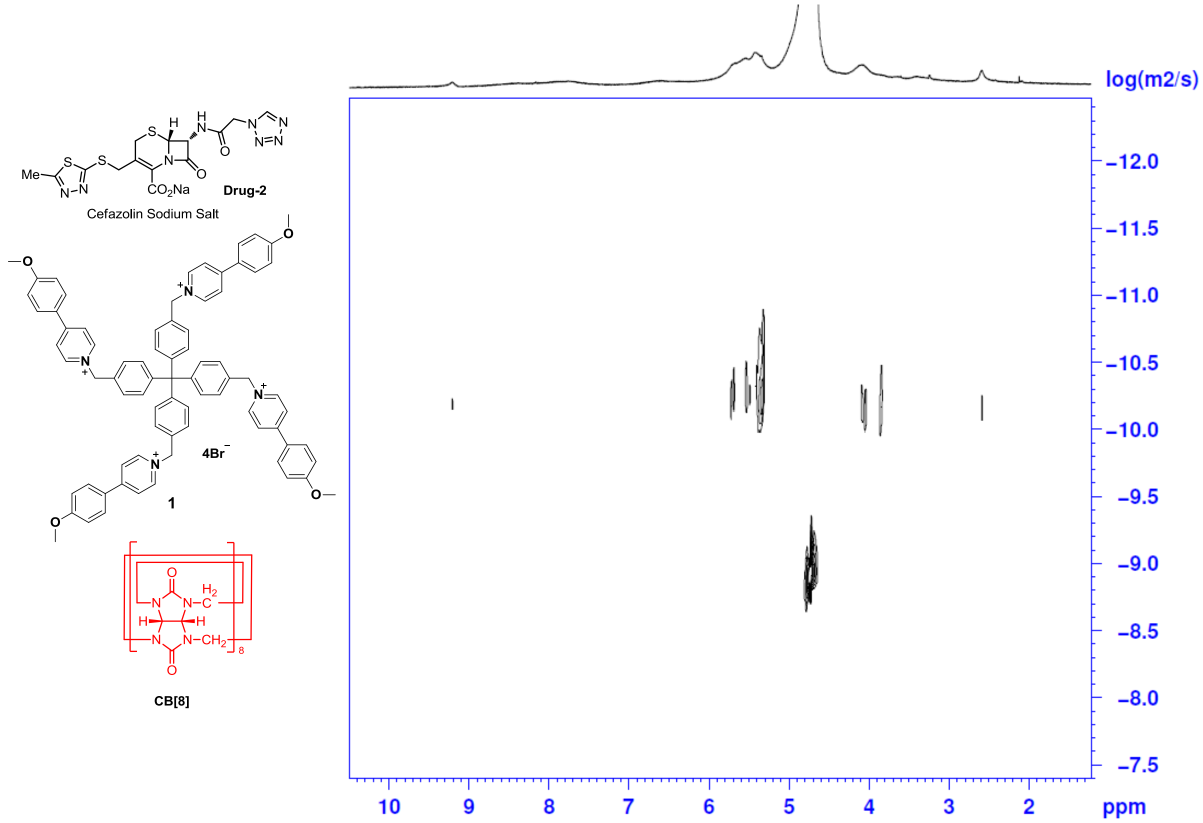

Supplementary Figure 19 | DOSY-^{1}H NMR spectrum (400 MHz). The solution of **1** (2 mM), CB[8] (4 mM) and **Drug-2** (8 mM) in D_2O. The ordinate represents the log value of the diffusion constant.

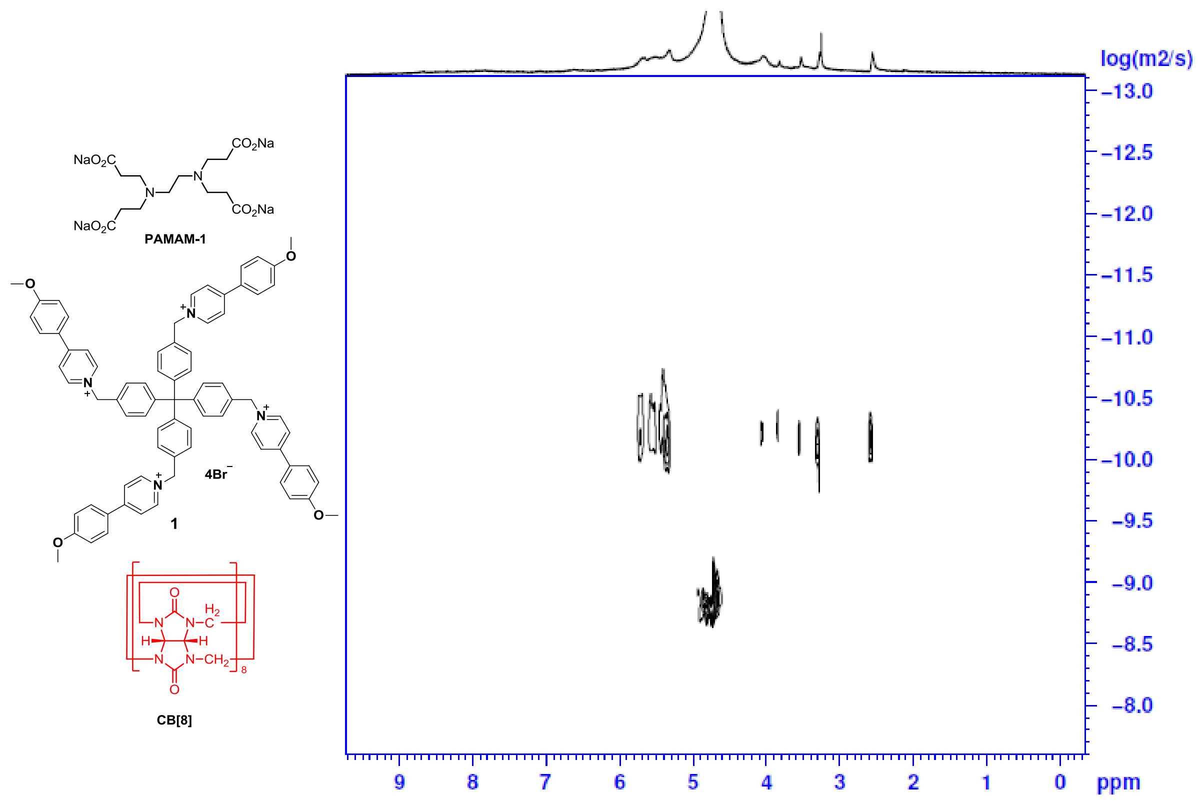

Supplementary Figure 20 | DOSY-^{1}H NMR spectrum (400 MHz). The solution of **1** (2 mM), CB[8] (4 mM) and **PAMAM-1** (2 mM) in D_2O. The ordinate represents the log value of the diffusion constant.

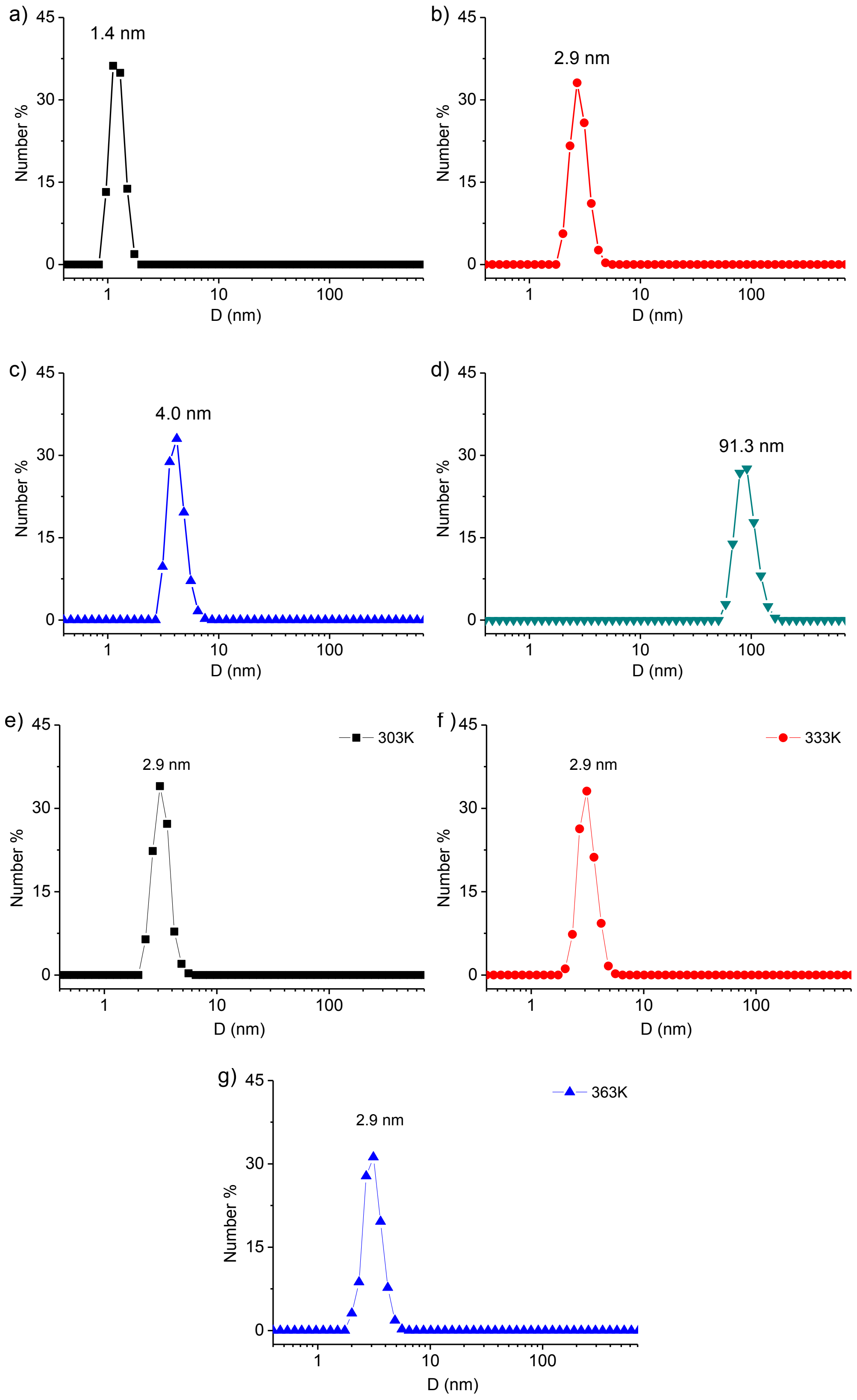

Supplementary Figure 21 | DLS results. The mixture solution of (a) **2**, (b) **3**, (c) **4**, (d) **1** and CB[8] in water at 25 °C, and the mixture solution of **3** and CB[8] at 30 °C (e), 60 °C (f) and 90 °C (g) ([PP] = 2[CB[8]] = 6.0 mM). The data represent the hydrodynamic diameters (D_H).

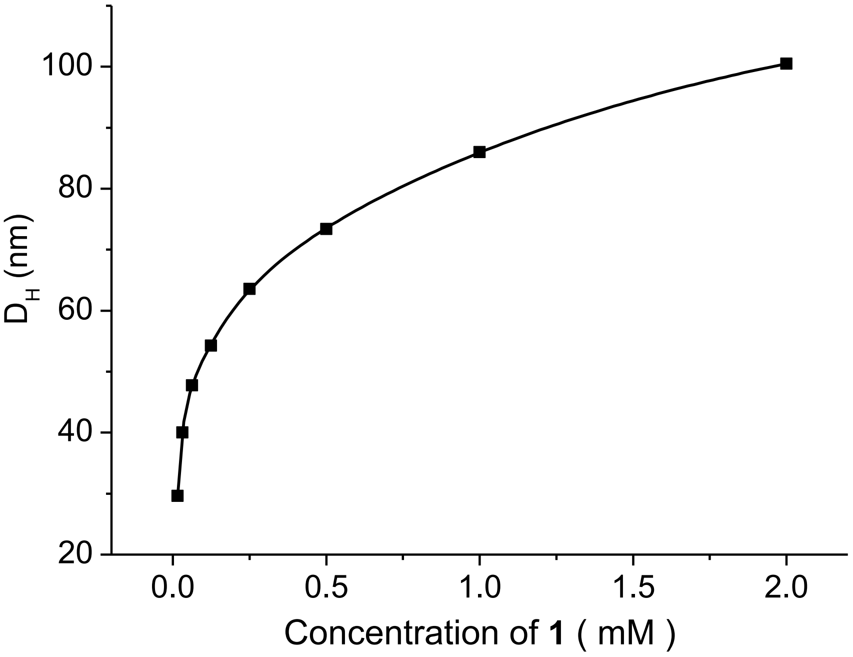

Supplementary Figure 22 | The concentration dependence of D_H. The aggregates of **1** and CB[8] (1:2) in water at 25 °C.

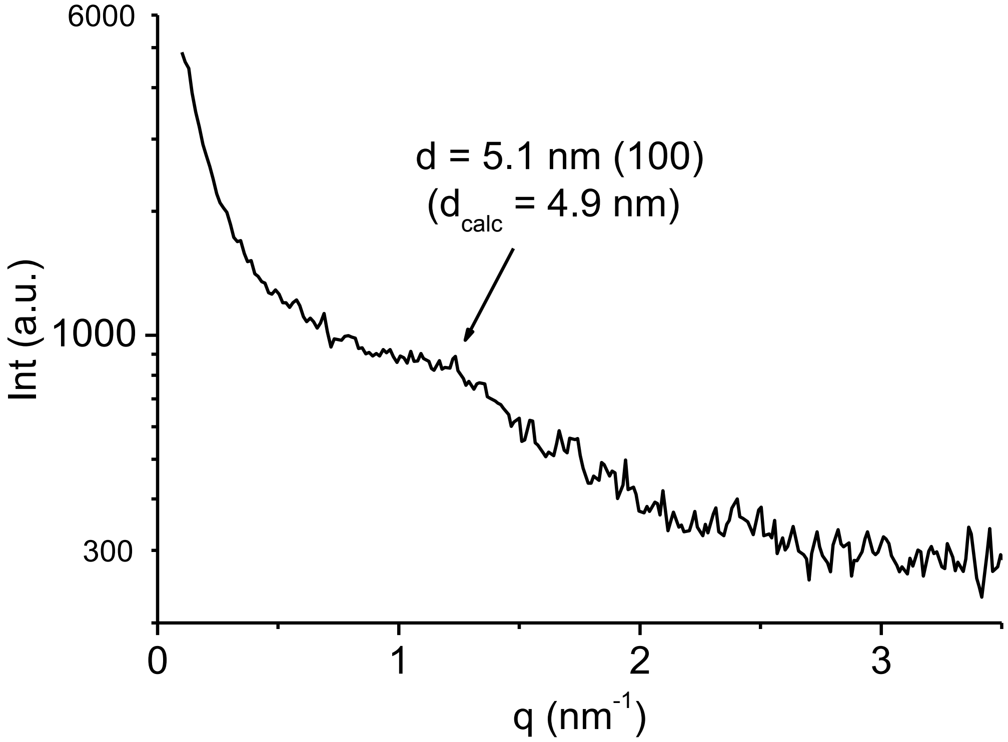

Supplementary Figure 23 | Solution-phase small-angle X-ray scattering profile. The 1:2 solution of **1** and CB[8] ([PP] = 2[CB[8]] = 8 mM) in water.

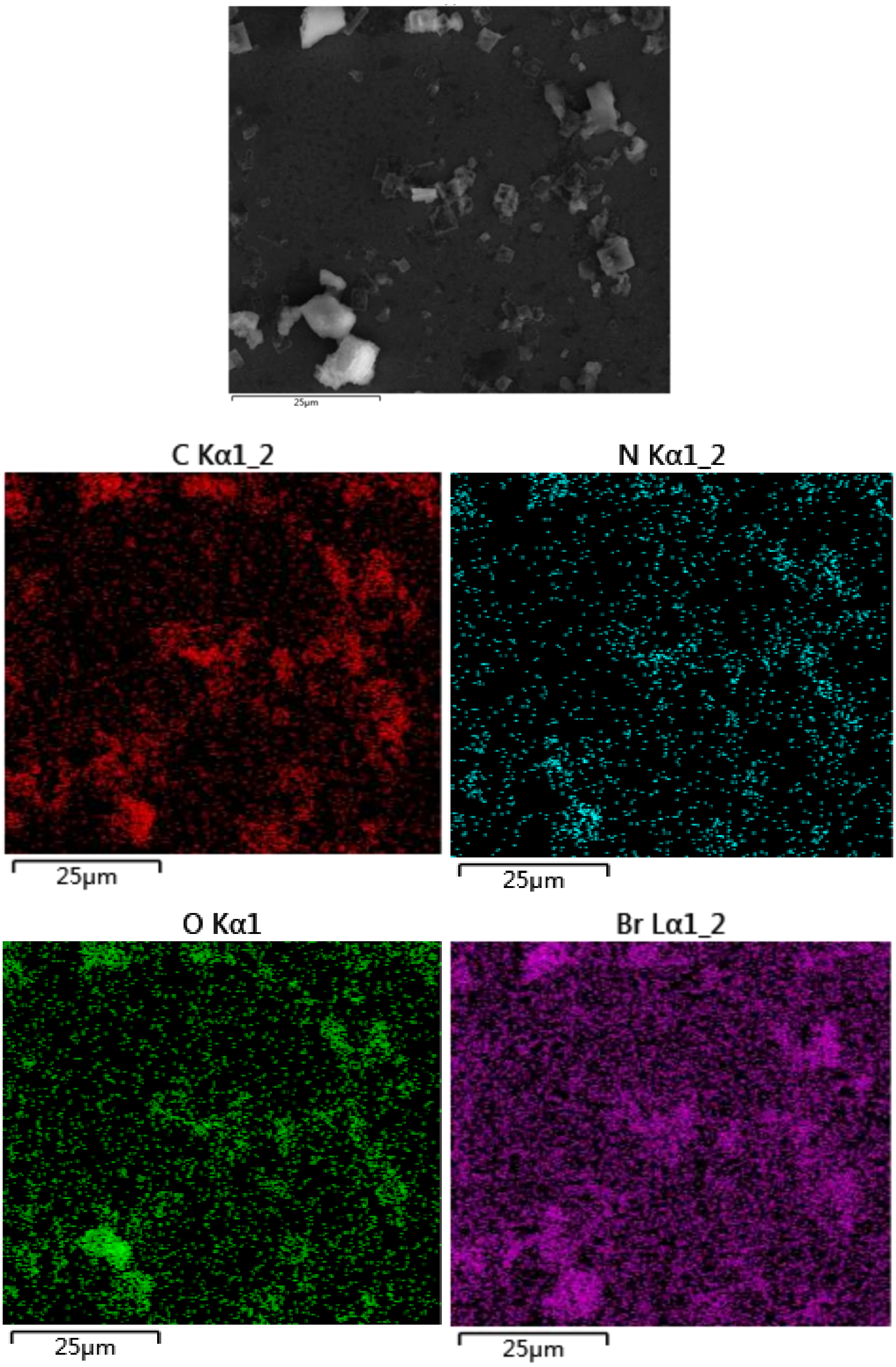

Supplementary Figure 24 | Element distribution mapping images. The microcrystals of the 3D SOF confirming the compositions of the C, N, O and Br elements by energy-dispersive X-ray spectroscopy.

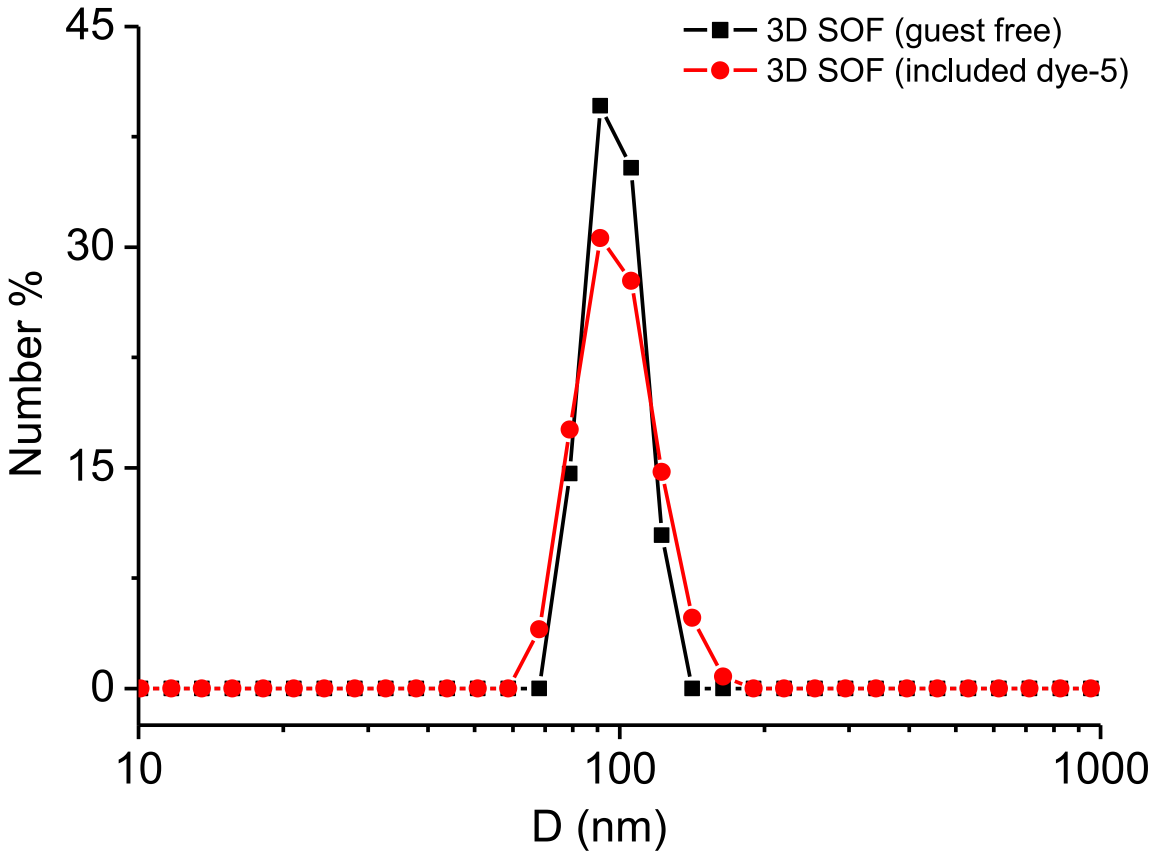

Supplementary Figure 25 | Comparison of DLS results. The solution of (guest free) 3D SOF (■) and (included **Dye-5**) 3D SOF (●) in water at 25 °C ([PP] = 2[CB[8]] = [anion] = 8.0 mM). The data represent the hydrodynamic diameters (D_H). [anion] represents the total concentration of the anionic unit of the **Dye-5**.

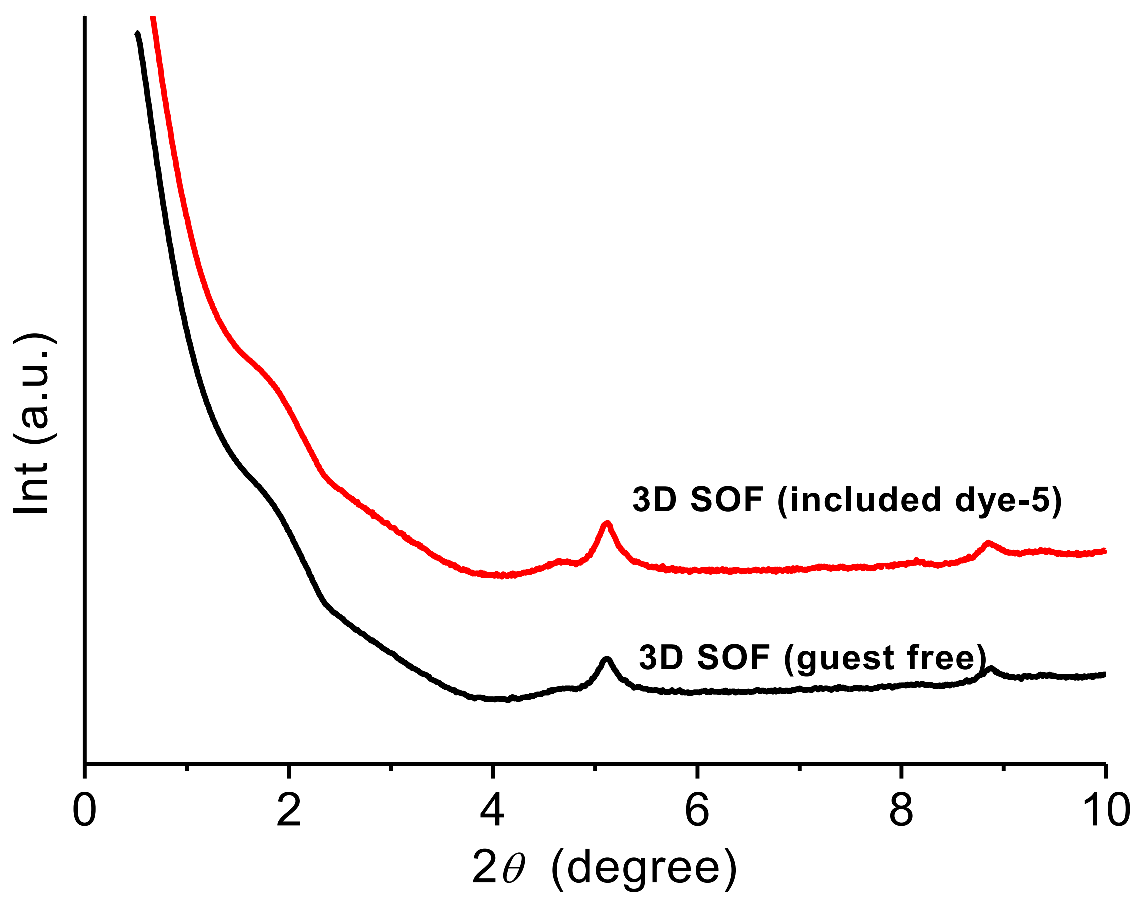

Supplementary Figure 26 | Comparison of the XRD profiles. Guest-free microcrystals of 3D SOF (black) and **Dye-5**-included microcrystals of 3D SOF (red).

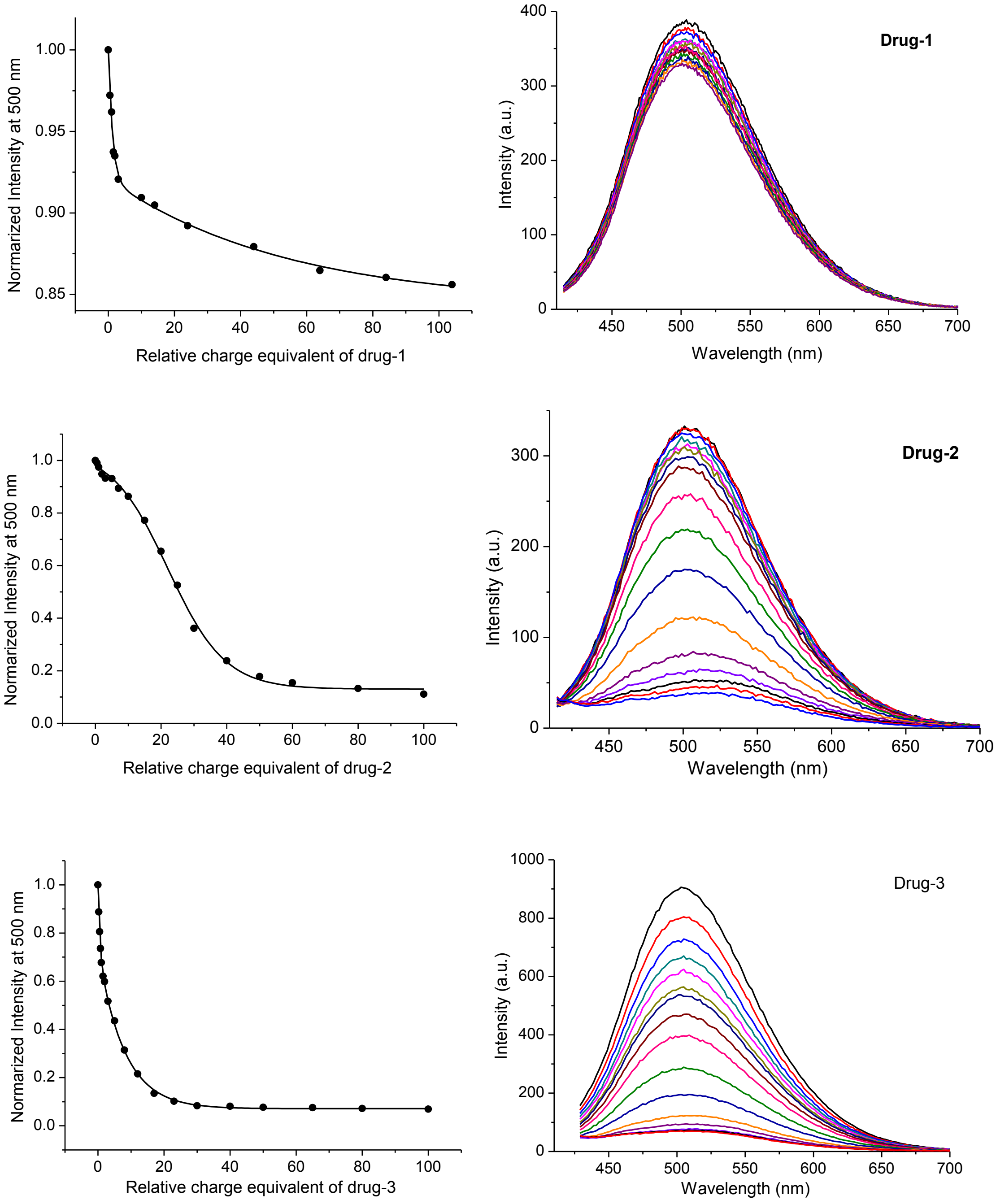

Supplementary Figure 27 | Fluorescence quenching. Drug-1~3 in **1**+CB[8] (1:2) mixture solution in water ([PP] = 2[CB[8]] = 0.02mM) by guests. This process was monitored through the decrease in fluorescence intensity at a selected wavelength as a function of relative charge equivalent. For each measurement, the initial absorbance was normalized to 1.0.

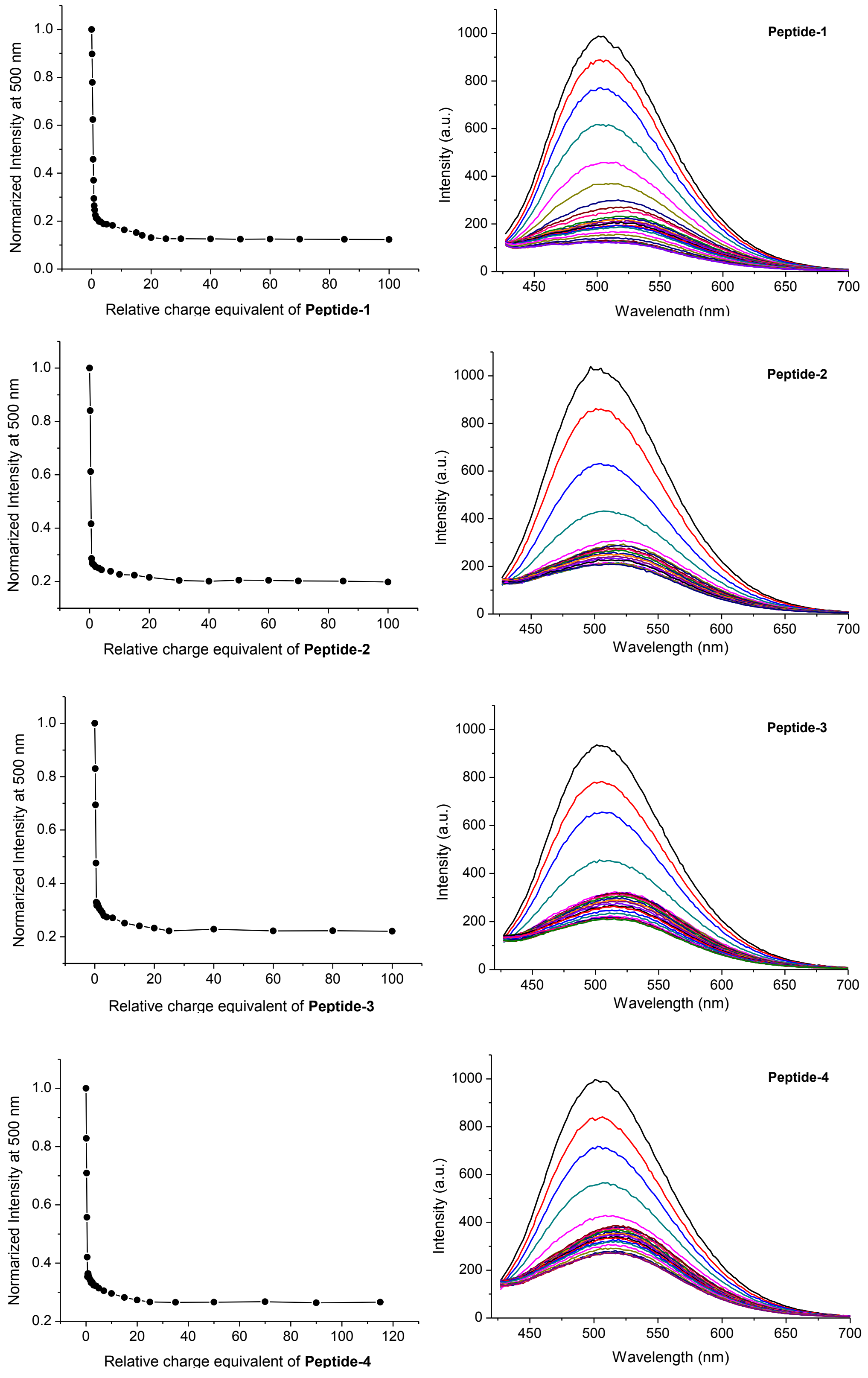

Supplementary Figure 28 | Fluorescence quenching. Peptide-1~4 in **1**+CB[8] (1:2) mixture solution in water ([PP] = 2[CB[8]] = 0.02 mM) by guests. This process was monitored through the decrease in fluorescence intensity at a selected wavelength as a function of relative charge equivalent. For each measurement, the initial absorbance was normalized to 1.0.

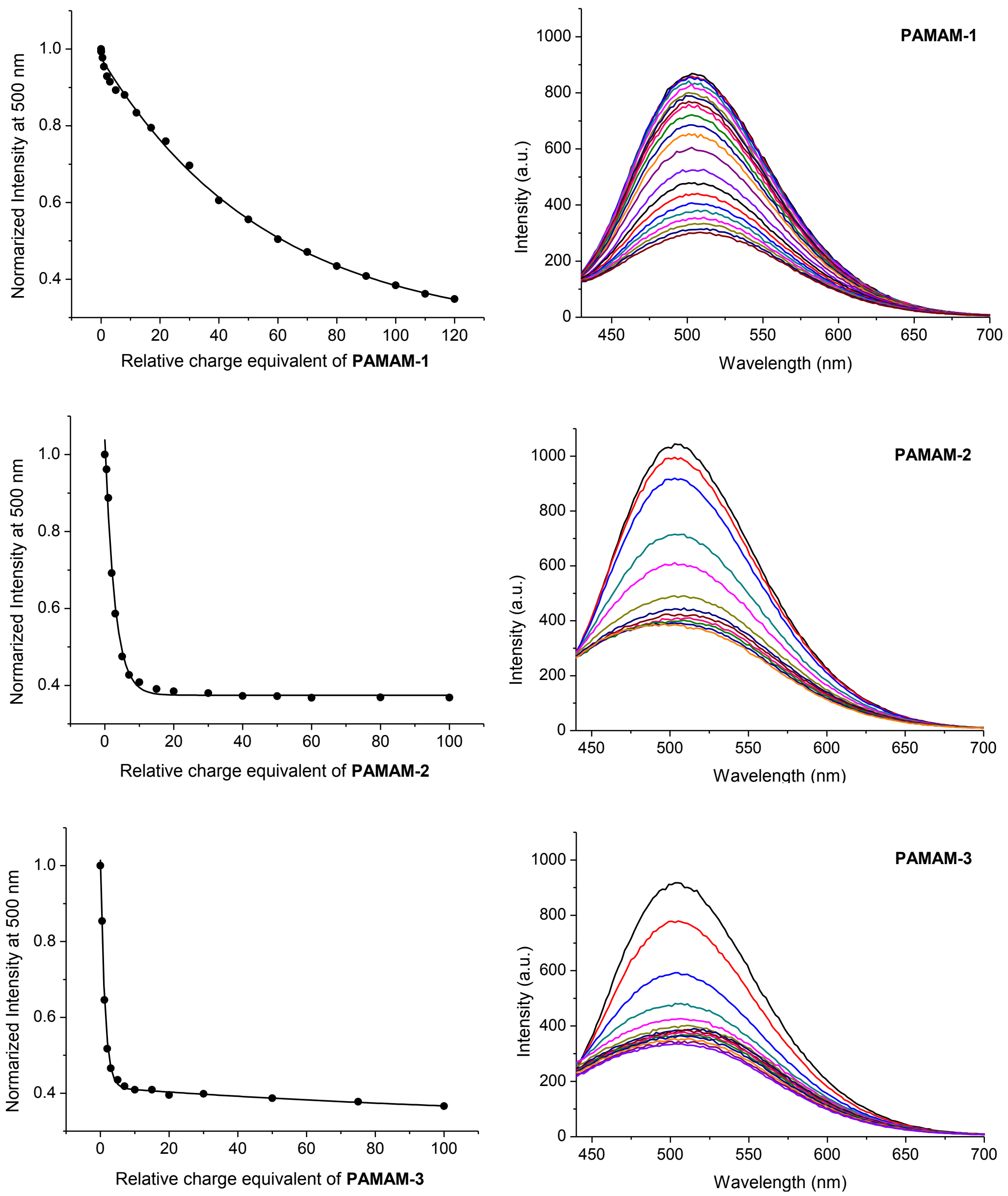

Supplementary Figure 29 | Fluorescence quenching. Dendrimers **PAMAM-1~3** in **1**+CB[8] 1:2 mixture solution in water ([PP] = 2[CB[8]] = 0.02mM) by guests. This process was monitored through the decrease in fluorescence intensity at a selected wavelength as a function of relative charge equivalent. For each measurement, the initial absorbance was normalized to 1.0.

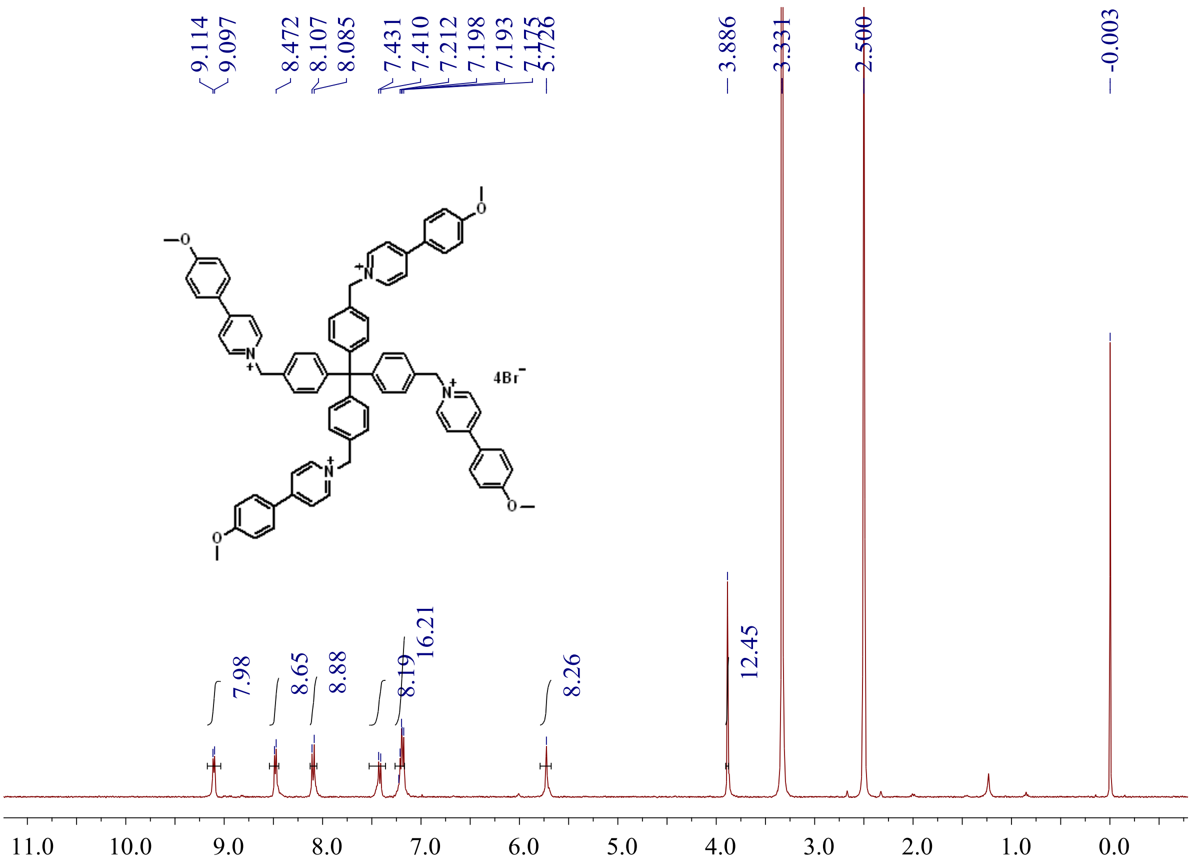

Supplementary Figure 30 | ^{1}H NMR spectrum (400 MHz). Compound **1** in DMSO-d_6 (4 mM).

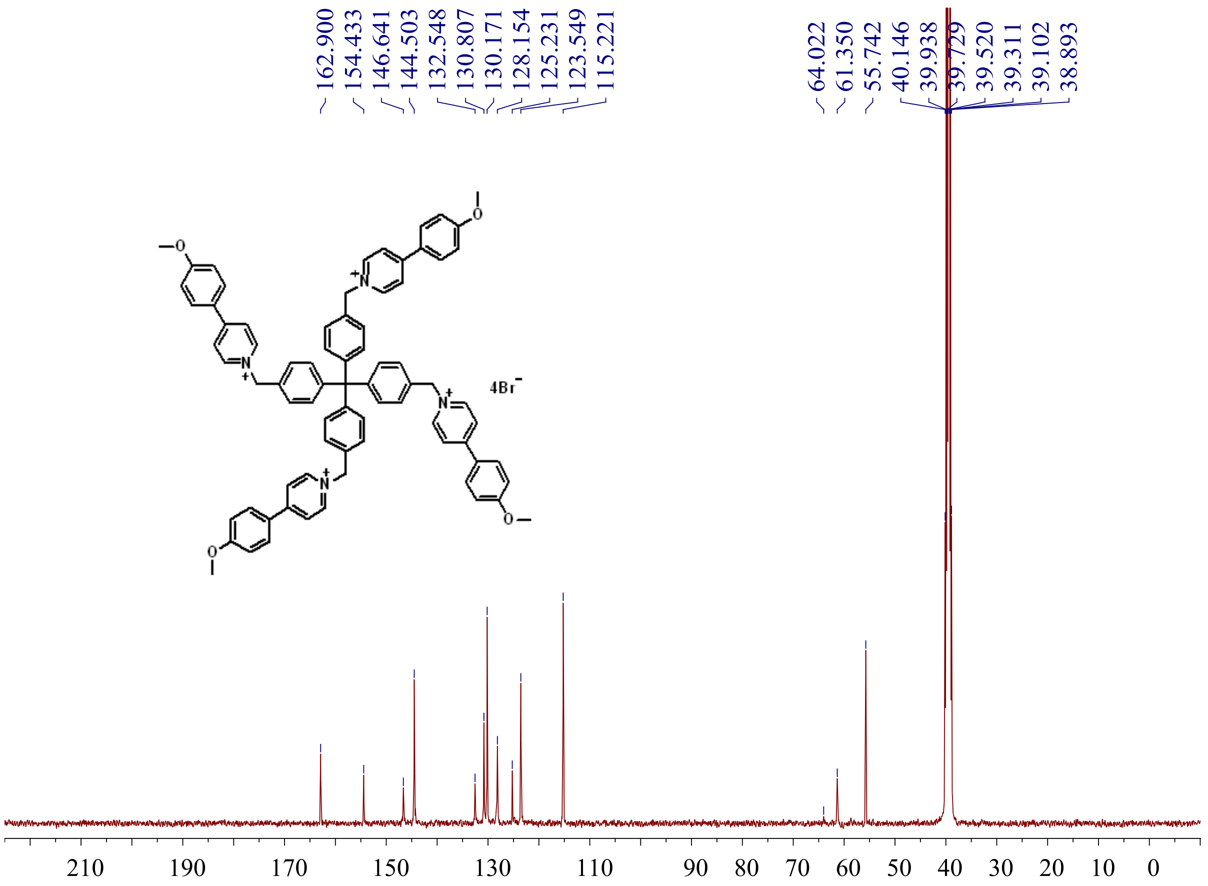

Supplementary Figure 31 | ^{13}C NMR spectrum (100 MHz). Compound **1** in DMSO-d_6 (10 mM).

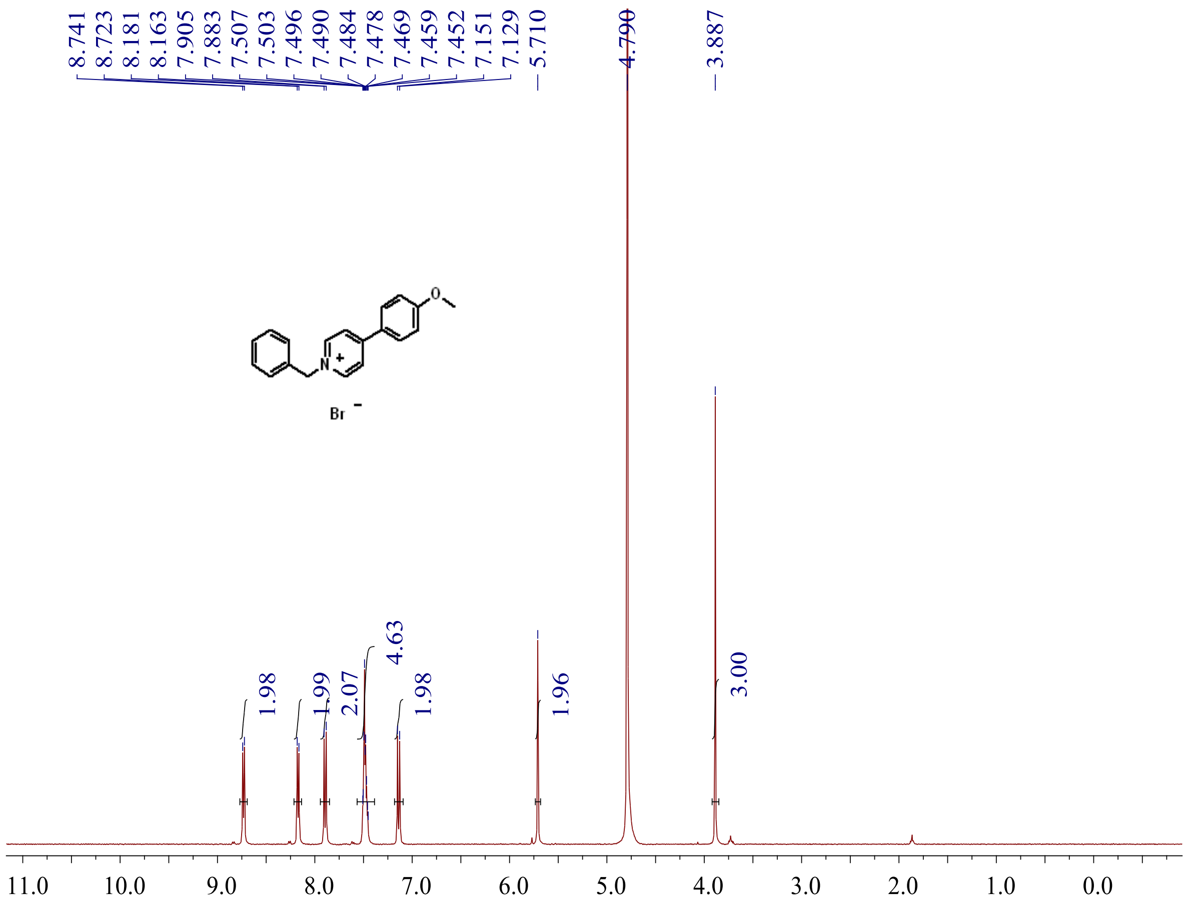

Supplementary Figure 32 | ^{1}H NMR spectrum (400 MHz). Compound **2** in D_2O (2 mM).

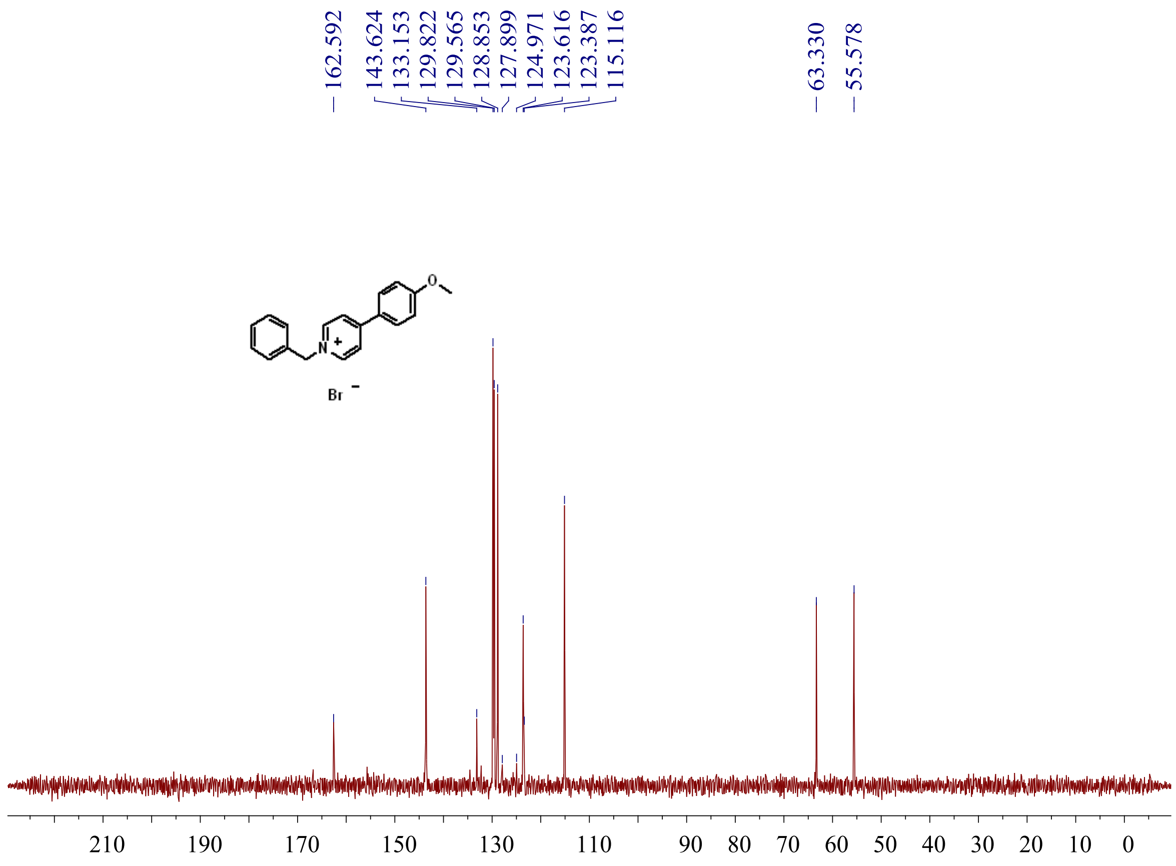

Supplementary Figure 33 | ^{13}C NMR spectrum (100 MHz). Compound **2** in D_2O (5 mM).

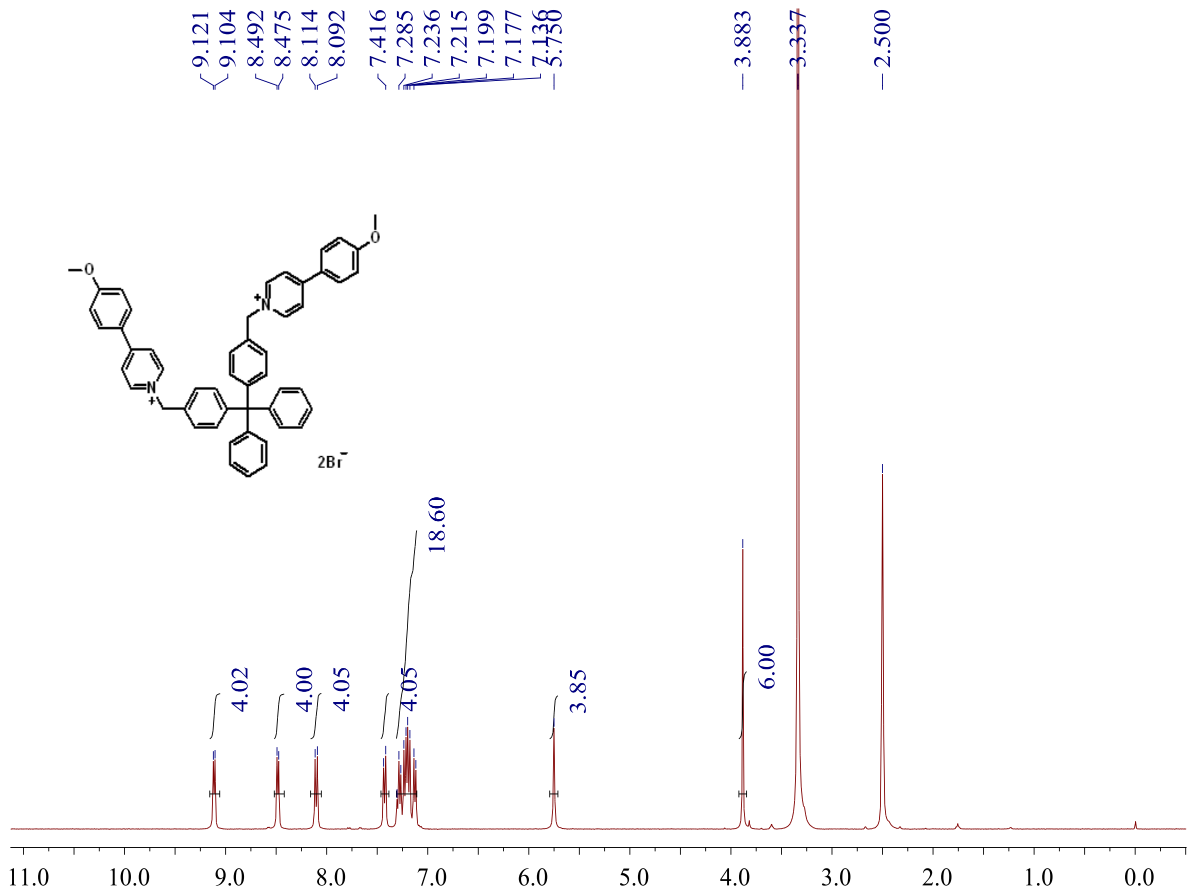

Supplementary Figure 34 | ^{1}H NMR spectrum (400 MHz). Compound **3** in DMSO-d_6 (3 mM).

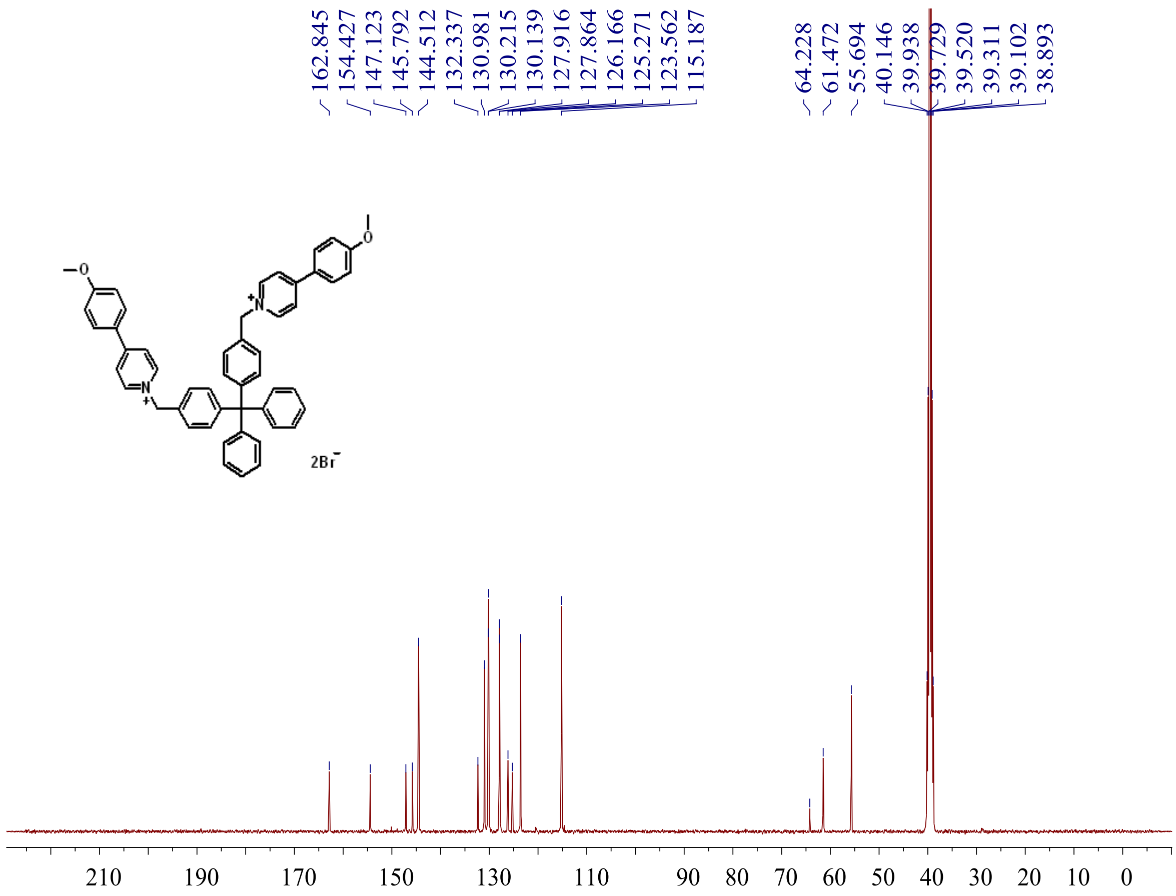

Supplementary Figure 35 | ^{13}C NMR spectrum (100 MHz). Compound **3** in DMSO-d_6 (10 mM).

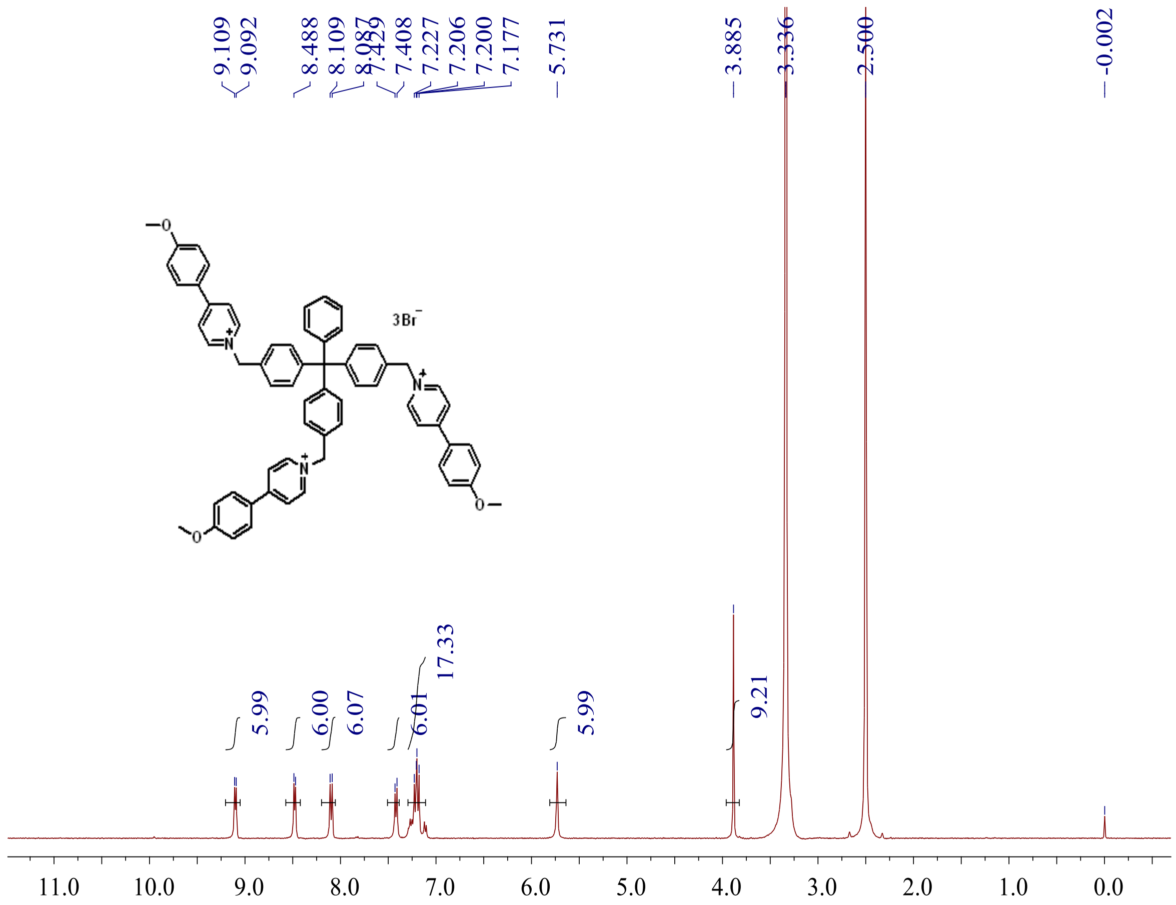

Supplementary Figure 36 | ^{1}H NMR spectrum (400 MHz). Compound **4** in DMSO-d_6 (3 mM).

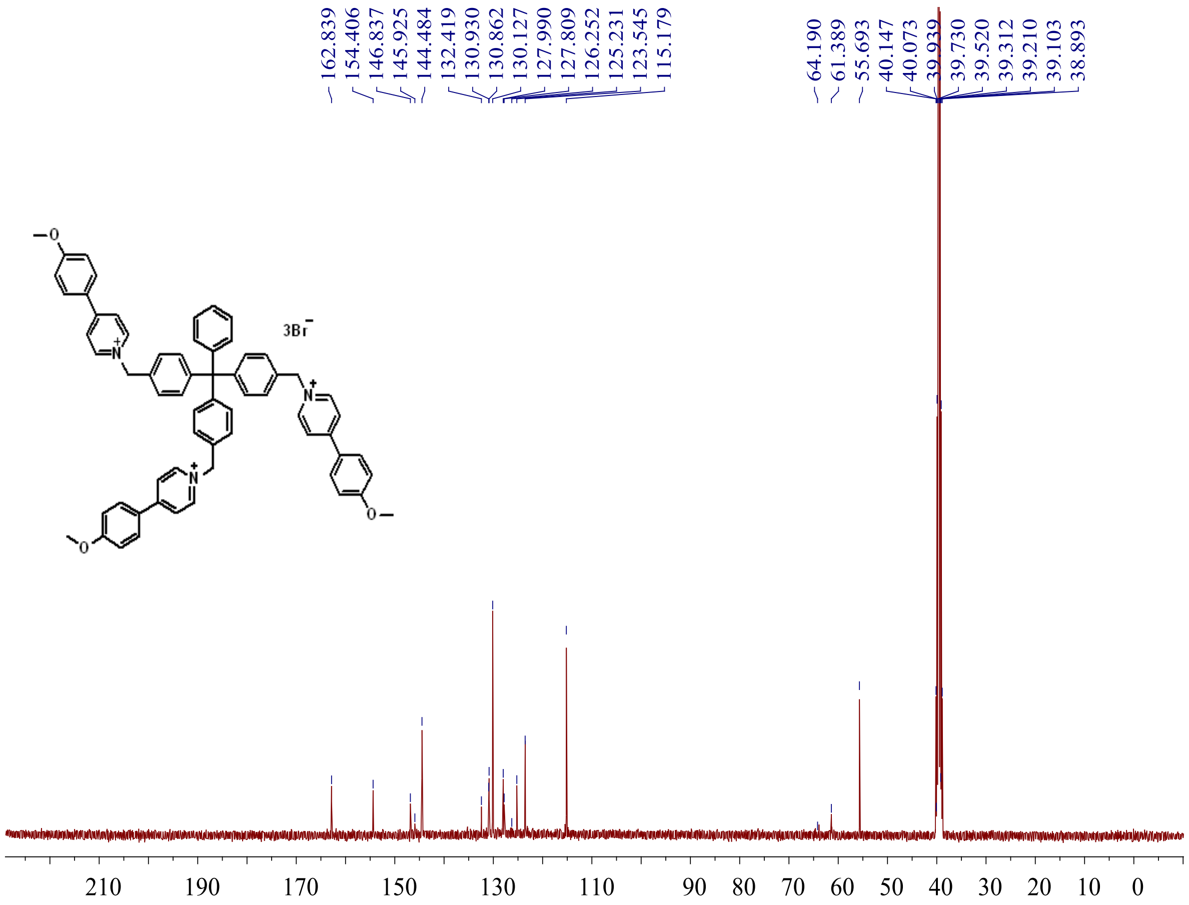

Supplementary Figure 37 | ^{13}C NMR spectrum (100 MHz). Compound **4** in DMSO-d_6 (8 mM).

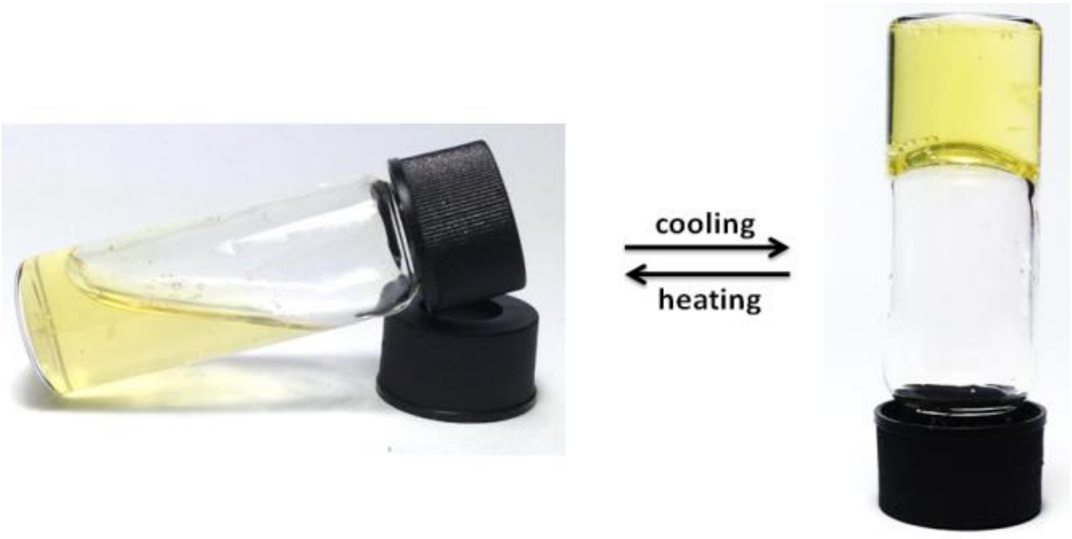

Supplementary Figure 38 | Illustration of the gel–solution transition. 3D SOF in water ([PP] = 2[CB[8]] = 10 mM). Left: 90 °C, right: 25 °C.

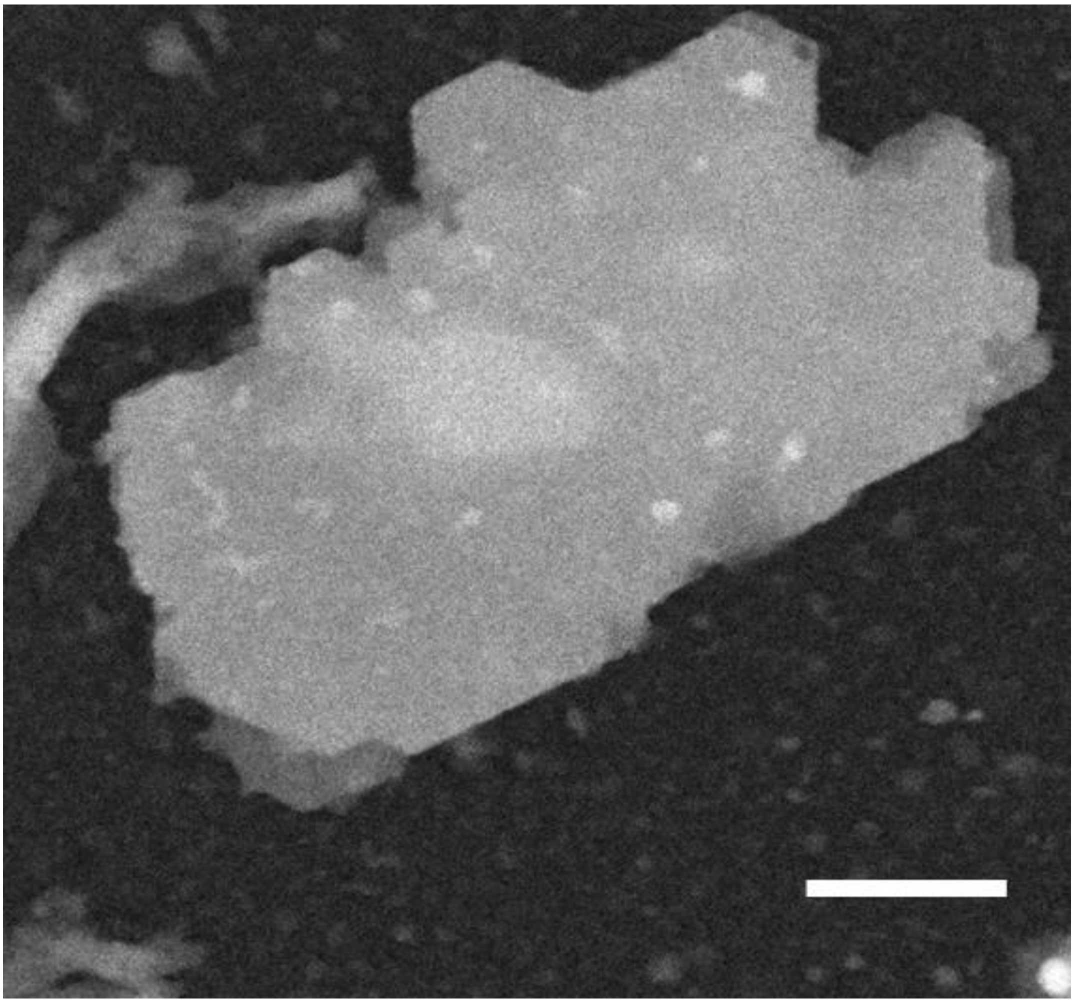

Supplementary Figure 39 | Cryo-TEM image. The species in Supplementary Figure 4f with no added white lines (bar width = 0.3 μm).

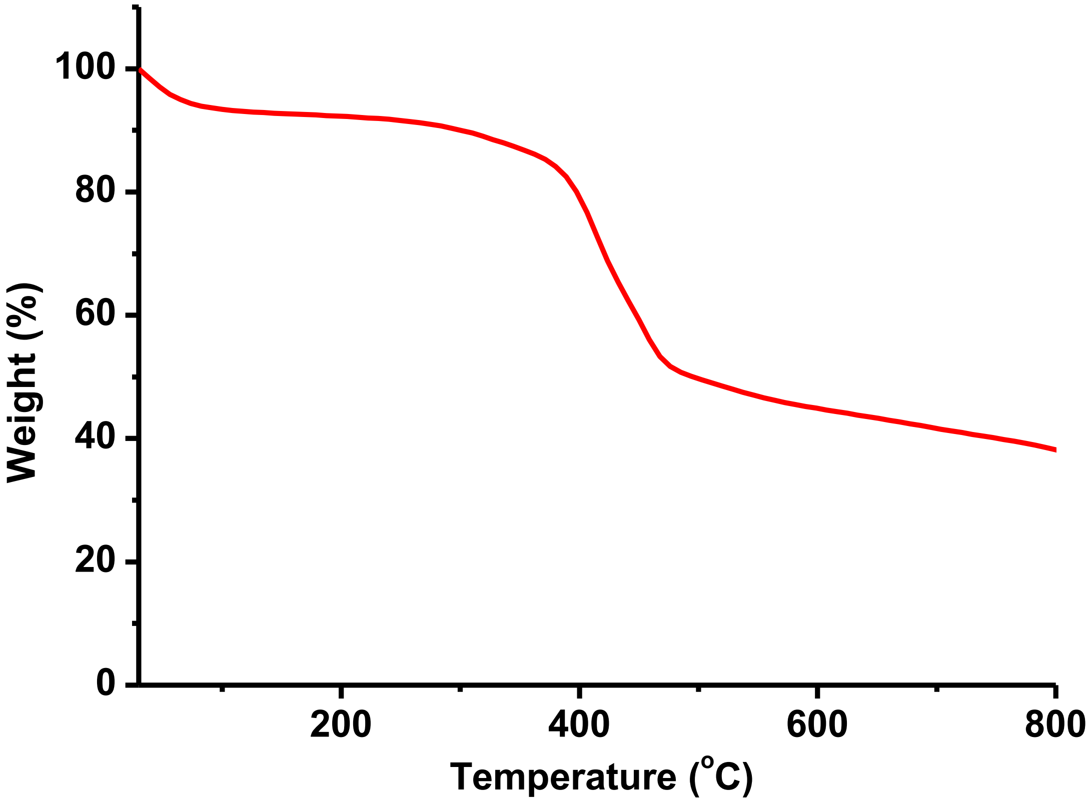

Supplementary Figure 40 | TGA trace. Microcrystals of 3D SOF.

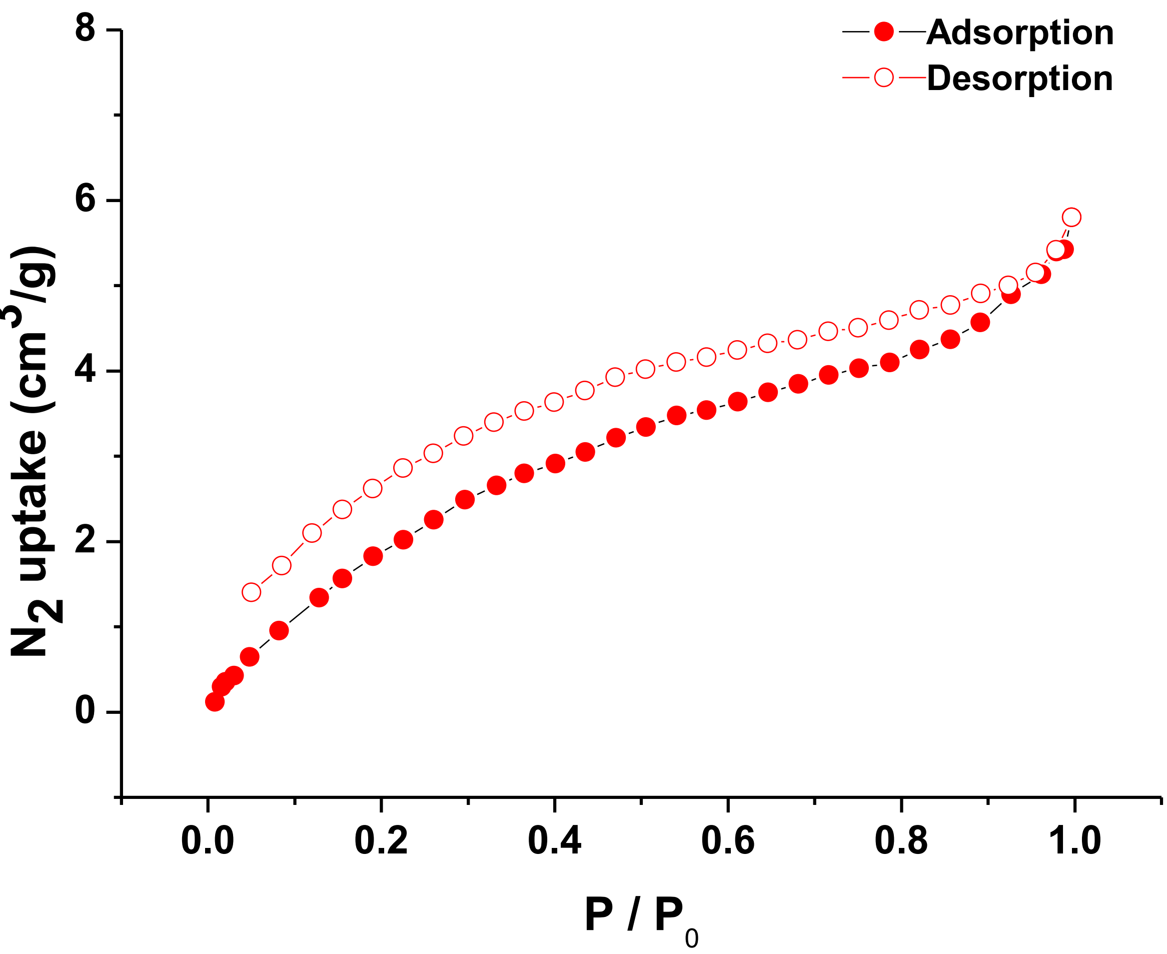

Supplementary Figure 41 | Nitrogen gas adsorption isotherm. 3D SOF at –196 °C.

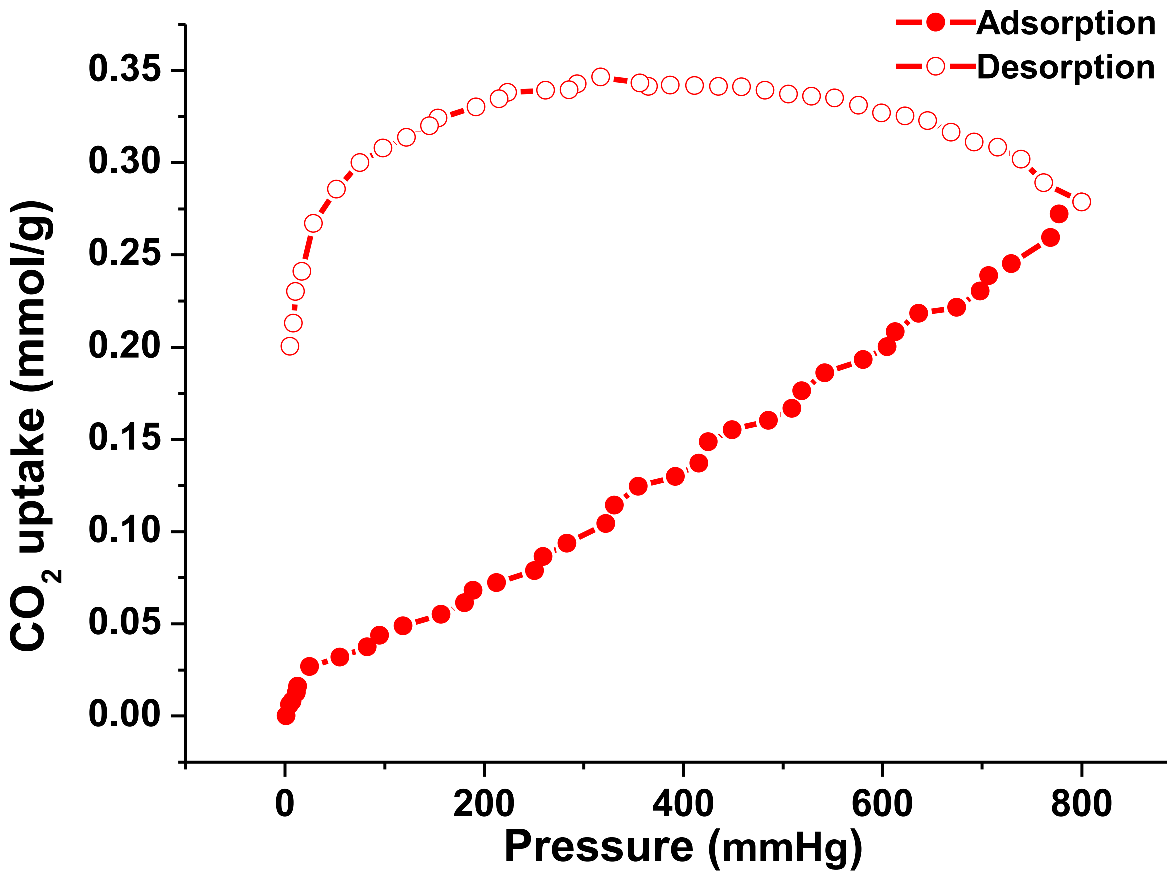

Supplementary Figure 42 | Carbon dioxide gas adsorption isotherm. 3D SOF at 0 °C.

Supplementary Methods

1. Materials

Dye-4 (anthraquinone-1,5-disulfonic acid disodium salt, purity: >98%), **Drug-3** (diclofenac sodium salt, purity: >98%) and **Drug-2** (Cefazolin sodium salt, purity: >97%) were purchased from Tokyo Chemical Industry Co., Ltd. **Dye-3** (sodium anthraquinone-2-sulfonate, purity: >98%), **PAMAM-1** (purity: >96%), **PAMAM-2** (purity: >96%, 20 wt.% in methanol), **PAMAM-3** (purity: >96%, 20 wt.% in methanol), **Dye-6** (1,3,6,8-pyrenetetrasulfonic acid tetrasodium salt hydrate, purity: >98%), **Dye-5** (8-hydroxypyrene-1,3,6-trisulfonic acid trisodium salt, purity: >97%), **Drug-1** (Adefovir, purity: >98%), **Dye-2** (2,6-naphthalenedisulfonic acid disodium salt, purity: >97%) and **Dye-1** (sodium benzenesulfonate, purity: >97%) were purchased from Sigma Aldrich Chemical Co. **Peptide-1** (purity: >99%), **Peptide-2** (purity: >99%), **Peptide-3** (purity: >99%), **Peptide-4** (purity: >99%), **sDNA-1** (purity: >99%), **sDNA-2** (purity: >99%), **sDNA-3** (purity: >99%), **sDNA-4** (purity: >99%), **DNA-1** (purity: >99%) and **DNA-2** (purity: >99%) were purchased from Sangon Biotech (Shanghai) Co., Ltd. Cucurbit[8]uril (CB[8]) hydrate (purity: 99+%) and 1-adamantanamine hydrochloride (purity: 99%) were purchased from J&K Scientific Co. Benzyl bromide (purity: 98%) was purchased from Sigma-Aldrich Chemical Co. Deuterium oxide (purity: 99.8 atom % D), dimethyl sulfoxide-d_6 (purity: 99.5 atom % D), acetonitrile (purity: 99.5%), tetrahydrofuran (purity: >99.0%), and acetone (purity: >99.0%) were purchased from Sigma-Aldrich Chemical Co. Water (CHROMASOLV® Plus, for HPLC) for preparing the samples for fluorescence, absorption, XRD, SAXS, TEM, SEM, and DLS experiments was purchased from Sigma Aldrich Chemical Co. Ultra-high-purity grade nitrogen gas (purity: 99.999%) and carbon dioxide gas (purity: 99.999%) for adsorption experiments were purchased from Shanghai Delun Gas Equipment Co. Compounds **5**[1], **6**[2], **7**[2], and **8**[2] were prepared according to reported procedures.

HeLa (human cervical carcinoma cell) lines were obtained from the cell bank of type culture collection of the Chinese Academy of Sciences (CBTCCCAS, Shanghai, China) and cultured at 37 °C in Dulbecco's modified Eagle medium (DMEM) supplemented with 10% fetal bovine serum (FBS), 2 mM L-glutamine, 100 μg/mL penicillin, and 50 μg/mL streptomycin in a 5% CO_2 environment.

2. Synthesis and characterizations

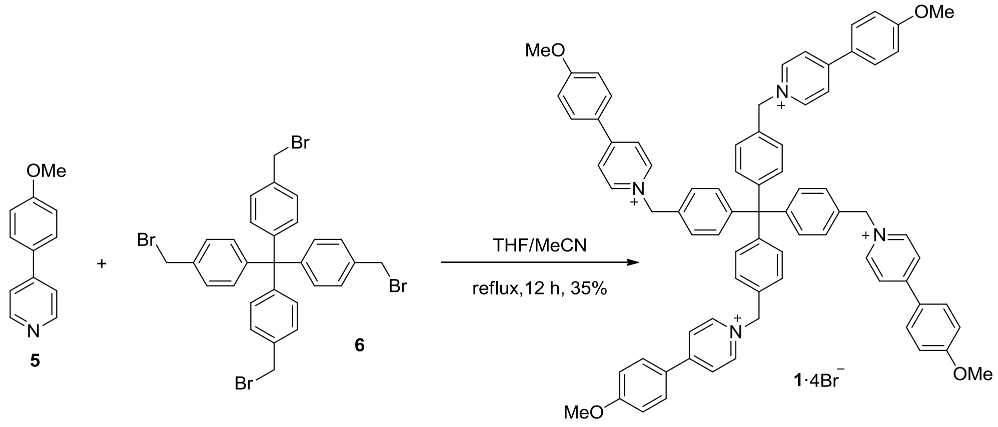

Compound 1·4Br$^-$**.** A mixture of compounds **5**[1] (0.19 g, 1.00 mmol) and **6**[2] (0.18 g, 0.25 mmol) in THF (5 mL) and acetonitrile (10 mL) was stirred under reflux for 12 h and then cooled to room temperature. To the mixture was added acetone (20 mL) and the resulting precipitate was filtrated and washed with acetone and dried under vacuum. The solid was further recrystallized from acetonitrile to give compound **1**·4Br$^-$ as a light yellow solid (0.13 g, 35%). M.p. > 300 °C (decomp). ^{1}H NMR (400 MHz, DMSO-d$_6$): δ 9.11 (d, *J* = 6.6 Hz, 8H), 8.48 (d, *J* = 6.9 Hz, 8H), 8.10 (d, *J* = 8.4 Hz, 8H), 7.42 (d, *J* = 8.4 Hz, 8H), 7.30-7.08 (m, 16H), 5.73 (s, 8H), 3.89 (s, 12H). ^{13}C NMR (100 MHz, DMSO-d$_6$): δ 162.9, 154.4, 146.6, 144.5, 132.6, 130.8, 130.2, 128.2, 125.2, 123.6, 115.2, 64.0, 61.4, 55.7. MS (ESI): *m/z* 635.2 [M-2Br]$^{2+}$. HRMS (ESI): Calcd for $C_{77}H_{68}Br_2N_4O_4$: 635.1798 [M-2Br]$^{2+}$. Found: 635.1786.

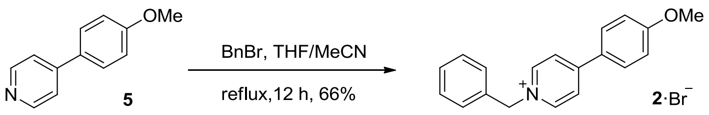

Compound 2·Br$^-$**.** A mixture of benzyl bromide (85 mg, 0.50 mmol) and **5** (93 mg, 0.15 mmol) in 12 mL of a mixture of THF (4 mL) and acetonitrile (8 mL) was refluxed for 12 h. After cooling to r.t, the reaction mixture was poured into acetone (20 mL). The formed precipitate was filtrated, and washed with acetone, and dried under vacuum to afford **2**·Br$^-$ as a white solid (0.12 g, 66%). M.p. >300 °C (decomp). ^{1}H NMR (400 MHz, D_2O): δ 8.73 (d, *J* = 7.0 Hz, 2H), 8.17 (d, *J* = 7.1 Hz, 2H), 7.89 (d, *J* = 9.0 Hz, 2H), 7.57-7.39 (m, 5H), 7.14 (d, *J* = 9.0 Hz, 2H), 5.71 (s, 2H), 3.89 (s, 3H). ^{13}C NMR (100 MHz, D_2O): δ 162.6, 143.6, 133.2, 129.8, 129.6, 128.9, 127.9, 125.0, 123.6, 123.4, 115.1, 63.3, 55.6. MS (ESI): *m/z* 276.1 [M–Br]$^+$. HRMS (ESI): Calcd for $C_{19}H_{18}NO$: 276.1383 [M–Br]$^+$. Found: 276.1398.

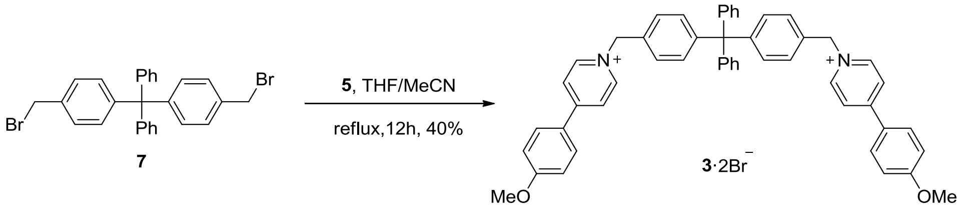

Compound 3·2Br$^-$ was prepared in 40% yield as a light yellow solid from the reaction of

compounds **5** and **7**[2] according to a procedure as described for **2**·Br^-. M.p. > 300 °C (decomp). ^{1}H NMR (400 MHz, DMSO-d_6): δ 9.11 (d, J = 6.8 Hz, 4H), 8.48 (d, J = 6.8 Hz, 4H), 8.10 (d, J = 8.9 Hz, 4H), 7.43 (d, J = 8.3 Hz, 4H), 7.31-7.11 (m, 18H), 5.75 (s, 4H), 3.88 (s, 6H). ^{13}C NMR (100 MHz, DMSO-d_6): δ 162.9, 154.4, 147.1, 145.8, 144.5, 132.3, 131.0, 130.2, 130.1, 127.9, 127.8, 126.2, 125.3, 123.6, 115.2, 64.2, 61.5, 55.7. MS (ESI): *m/z* 795.2 $[M-Br]^+$. HRMS (ESI): Calcd for $C_{51}H_{44}BrN_2O_2$: 795.2581 $[M-Br]^+$. Found: 795.2570.

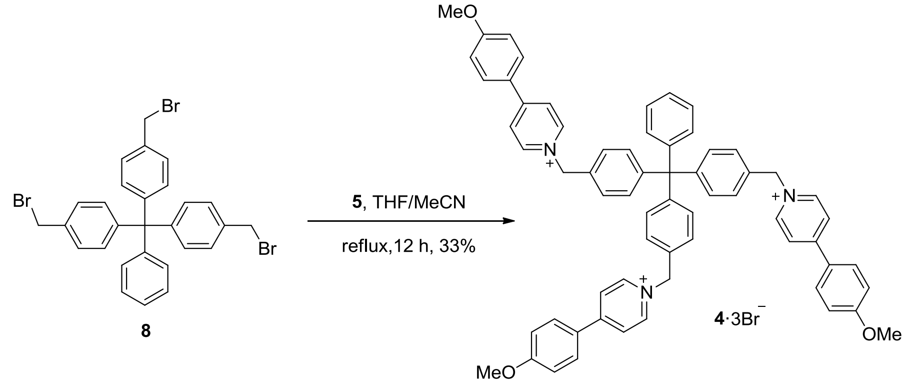

Compound 4·3Br^- was prepared in 33% yield as a light yellow solid from the reaction of compounds **5** and **8**[2] according to a procedure as described for **2**·Br^-. M.p. > 300 °C (decomp). ^{1}H NMR (400 MHz, DMSO-d_6): δ 9.10 (d, J = 6.7 Hz, 6H), 8.48 (d, J = 6.8 Hz, 6H), 8.10 (d, J = 8.4 Hz, 6H), 7.42 (d, J = 8.4 Hz, 6H), 7.43-7.18 (m, 17H), 5.73 (s, 6H), 3.88 (s, 9H). ^{13}C NMR (100 MHz, DMSO-d_6): δ 162.8, 154.4, 146.8, 145.9, 144.5, 132.4, 130.9, 130.8, 130.1, 128.0, 127.8, 126.3, 125.2, 123.6, 115.2, 64.2, 61.4, 55.7. MS (ESI): *m/z* 1072.2 $[M-Br]^+$. HRMS (ESI): Calcd for $C_{64}H_{56}Br_2N_3O_3$: 1072.2683 $[M-Br]^+$. Found: 1072.2668.

3. The method for determining the apparent association constants.

The concentration of CB[8] and the concentration of the PP unit of the investigated compounds were kept at 1:2. The thermodynamics of the host-guest complexation and competition experiments were defined as the following equations:[3]

$$\text{CB[8]} + 2\text{PP} \xrightleftharpoons{K_a} \text{CB[8]}\subset 2\text{PP} \quad (1)$$

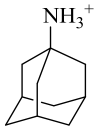

$$\text{CB[8]} + \text{AD} \xrightleftharpoons{K_{AD}} \text{CB[8]}\subset\text{AD} \quad (2)$$

AD

$$\text{CB[8]}\subset 2\text{PP} + \text{AD} \xrightleftharpoons{K_{rel}} \text{CB[8]}\subset\text{AD} + 2\text{PP} \quad (3)$$

For compounds **1**, **3** and **4**, their complexes with CB[8] exhibited a set of new signals. The following equation was used to determine K_a:

$$K_{rel} = 4C^0_{CB[8]\subset 2PP}\frac{x^3_{free}}{(1-x_{free})\left(C^0_{AD}/C^0_{CB[8]\subset 2PP} - x_{free}\right)} \quad (4)$$

Where $K_{rel} = K_{AD}/K_a$, $C^0_{CB[8]\subset 2PP}$ was the concentration of the 2:1 complex of PP and CB[8], C^0_{AD} was the total concentration of guest 1-adamantanamine hydrochloride, x_{free} was the ratio of the concentration of the free PP unit over the total concentration of the PP unit.

For compound **2** the chemical shifts of its signals changed upon complexation by CB[8]. The following equations were used to determine K_a:

$$\delta = \delta_{free} \times x_{free} + \delta_{complex} \times x_{complex} \tag{5}$$

$$K_{rel} = 4C^0_{CB[8]\subset 2MOPP} \frac{\left(\dfrac{\delta - \delta_{complex}}{\delta_{free} - \delta_{complex}}\right)^3}{(\dfrac{\delta_{free} - \delta}{\delta_{free} - \delta_{complex}})(C^0_{AD}/C^0_{CB[8]\subset 2MOPP} - \dfrac{\delta - \delta_{complex}}{\delta_{free} - \delta_{complex}})} \tag{6}$$

Where $K_{rel} = K_{AD}/K_a$, $C^0_{CB[8]\subset 2PP}$ was the concentration of the 2:1 complex of the PP unit and CB[8], C^0_{AD} was the concentration of 1-adamantanamine hydrochloride, $\delta_{complex}$ represents the chemical shifting of the signal of the PP unit in the complex, δ_{free} represents the chemical shifting of the signal of the free PP unit, x_{free} was the ratio of the concentration of the free PP unit, $x_{complex}$ was the ratio of the concentration of the PP unit in the complex. K_{AD} had been previously determined to be 8.2×10^8 M^{-1} in 50 mM CD_3CO_2Na buffer (pD = 4.74).

4. Dynamic light scattering (DLS) measurement

DLS data were obtained on a Malvern Zetasizer Nano ZS90 using a monochromatic coherent He–Ne laser (633 nm) as the light source and a detector that detected the scattered light at an angle of 90 °.

5. UV-Vis measurement procedure

UV-vis spectra were performed on a Perkin-Elmer 750s instrument. Activated microcrystals (1.0 mg) were added to a solution of anionic guests in water (1 mL). The molar quantity of the anionic unit of all the guests was equal to that of the PP unit in **1** of the microcrystals. The UV-vis spectra of the solution were recorded after different standing time (up to 60 h). The decreased absorption of the guests was then used to calculate the amount of the guests that were absorbed into the interior of the microcrystals.

6. TGA measurement

TGA experiments were performed on a Model TGA/SDTA 851 instrument. Samples were placed in alumina pans and heated at a rate of 5 °C per minute from 30 to 800 °C under a nitrogen atmosphere.

7. Cytotoxicity test

Microcrystals of **Com-Tetra** (50 mg) in **Drug-1~3** (0.4 M, 10 mL, in water) solution were stirred at 25 °C for 6 d. The resultant solid was filtered, washed with water, and dried in vacuo to yield **Drug-1~3**-contained **Com-Tetra**. The *in vitro* cytotoxicity was measured using the Cell Counting Kit-8 (CCK-8) assay in human cervical carcinoma cell line HeLa. Cells

growing in log phase were seeded into 96-well cell-culture plate at 2 × 10^4/well and then incubated for 24 h at 37 °C under 5% CO_2. Phosphate buffer solution (PBS) of the **Com-Tetra** samples (10 μL/well) at concentrations of 50, 100, 150, or 200 μg /mL was added to the wells of the treatment group, and PBS (10 μL/well) to the negative control group, respectively. The cells were incubated for 24 h at 37 °C under 5% CO_2. Subsequently, 10 μL CCK-8 (1×) was added to each well of the 96 well assay plate and incubated for an additional 2 h at 37 °C under 5% CO_2. The absorbance (A value) of each well with background subtraction was measured at 450 nm. The following formula was used to calculate the viability of cell growth: Cell viability (%) = (mean of A value of treatment group/mean of A value of control) ×100.

8. Gas adsorption experiments

Gas adsorptions of N_2 at −196 °C and CO_2 at 0 °C were measured on a Micromeretics Model ASAP 2020 gas adsorption analyzer. About 88 mg of activated sample was degassed at 180 °C for 10 h by using the "outgas" function of the surface area analyzer. Helium gas was used to estimate the dead volume. The saturation pressure (P0) was measured throughout the N_2 analyses via a dedicated saturation pressure transducer, which helped to monitor the vapor pressure for each data point. Part of the N_2 sorption isotherm in the normalized pressure (P/P0) range of 0.05−0.3 was used to calculate the BET surface area. For CO_2 isotherm measurements, activated sample was transferred into a pre-weighed glass sample tube. The tube was then sealed and quickly transferred to a system providing 10^{-4} torr dynamic vacuum. The sample was kept under this vacuum at 180 °C for 10 h and then used for CO_2 adsorption measurements.

Supplementary References

1. Kitamura, Y. *et al.* Ligand-free Pd/C-catalyzed Suzuki–Miyaura coupling reaction for the synthesis of heterobiaryl derivatives. *Chem. Commun.* **47**, 5069-5071 (2007).
2. Tian, J. *et al*. Self-Assembly of Three-dimensional supramolecular polymers through cooperative tetrathiafulvalene radical cation dimerization. *Chem. Eur. J.* **20**, 575–584 (2014).
3. Liu, S. *et al.* The cucurbit[n]uril family: prime components for self-sorting systems. *J. Am. Chem. Soc*. **127**, 15959–15967 (2005).
